# Multiplatform molecular profiling uncovers two subgroups of malignant peripheral nerve sheath tumors with distinct therapeutic vulnerabilities

**DOI:** 10.1038/s41467-023-38432-6

**Published:** 2023-05-10

**Authors:** Suganth Suppiah, Sheila Mansouri, Yasin Mamatjan, Jeffrey C. Liu, Minu M. Bhunia, Vikas Patil, Prisni Rath, Bharati Mehani, Pardeep Heir, Severa Bunda, German L. Velez-Reyes, Olivia Singh, Nazanin Ijad, Neda Pirouzmand, Tatyana Dalcourt, Ying Meng, Shirin Karimi, Qingxia Wei, Farshad Nassiri, Trevor J. Pugh, Gary D. Bader, Kenneth D. Aldape, David A. Largaespada, Gelareh Zadeh

**Affiliations:** 1 MacFeeters-Hamilton Centre for Neuro-Oncology Research, Princess Margaret Cancer Centre, Toronto, ON Canada; 2 Division of Neurosurgery, Department of Neurosurgery, University of Toronto, Toronto, ON Canada; 3 Faculty of Science, Thompson Rivers University, Kamloops, BC Canada; 4 The Donnelly Centre, University of Toronto, Toronto, ON Canada; 5 Masonic Cancer Center, University of Minnesota, Minneapolis, MN USA; 6 Ontario Institute for Cancer Research, Toronto, ON Canada; 7 Laboratory of Pathology, Center for Cancer Research, National Cancer Institute, Bethesda, MD USA; 8 Department of Medical Biophysics, University of Toronto, Toronto, ON Canada; 9 Princess Margaret Cancer Centre, University Health Network, Toronto, ON Canada; 10 Department of Molecular Genetics, University of Toronto, Toronto, ON Canada; 11 Department of Computer Science, University of Toronto, Toronto, ON Canada; 12 Department of Pediatrics, University of Minnesota, Minneapolis, MN USA

**Keywords:** Sarcoma, Cancer genomics

## Abstract

Malignant peripheral nerve sheath tumor (MPNST) is a highly aggressive sarcoma, and a lethal neurofibromatosis type 1-related malignancy, with little progress made on treatment strategies. Here, we apply a multiplatform integrated molecular analysis on 108 tumors spanning the spectrum of peripheral nerve sheath tumors to identify candidate drivers of MPNST that can serve as therapeutic targets. Unsupervised analyses of methylome and transcriptome profiles identify two distinct subgroups of MPNSTs with unique targetable oncogenic programs. We establish two subgroups of MPNSTs: SHH pathway activation in MPNST-G1 and WNT/ß-catenin/CCND1 pathway activation in MPNST-G2. Single nuclei RNA sequencing characterizes the complex cellular architecture and demonstrate that malignant cells from MPNST-G1 and MPNST-G2 have neural crest-like and Schwann cell precursor-like cell characteristics, respectively. Further, in pre-clinical models of MPNST we confirm that inhibiting SHH pathway in MPNST-G1 prevent growth and malignant progression, providing the rational for investigating these treatments in clinical trials.

## Abstract (web summary)

Malignant peripheral nerve sheath tumours are an aggressive form of sarcoma, with limited treatment options. Here, the authors utilise DNA methylation and transcriptomic data to identify two subtypes of tumours with potential therapeutic vulnerabilities.

## Introduction

Malignant peripheral nerve sheath tumors (MPNSTs) represent a highly aggressive and lethal subtype of peripheral nerve sheath tumors (PNSTs) that confer a 5-year survival rate as low as 20–50%^1^. Approximately half of all MPNSTs occur in the setting of the hereditary neurofibromatosis type 1 (NF1) tumor predisposition syndrome, which afflicts 1 in 3000 individuals^2^. The hallmark of NF1 is the development of PNSTs that fall within a spectrum of benign (cutaneous and intraneural neurofibromas) and malignant (MPNST). Plexiform intraneural neurofibromas are present in around 30–50% of NF1 patients and harbor a 5–15% lifetime risk of malignant transformation^3^. Recently, atypical neurofibromas have been described with premalignant features consisting of increased cellularity, cytological atypia and/or a fascicular growth pattern^4,5^. On the malignant end of the spectrum, MPNSTs are sarcomas causing an average reduction in life expectancy by 5–10 years in the NF1 population^6^. Standard of care includes maximal surgical resection with adjuvant radiation therapy, with few effective chemotherapies presently available. Limited advances in the clinical outcome of these aggressive sarcomas reflect gaps in knowledge in the mechanism of malignant transformation and lack of candidates in the therapeutic pipeline.

The drivers of malignant transformation are not fully understood in PNSTs. In benign neurofibromas, the loss of *NF1*, which is a classic tumor suppressor gene that encodes neurofibromin and negatively regulates the RAS-MAPK (mitogen-activated protein kinase) pathway, occurs early^2^. Atypical neurofibromas are characterized by *CDKN2A/B* deletion in addition to *NF1* loss, suggesting this is the next step in tumor progression^4^. What is known in MPNSTs is that there is a loss of Polycomb repressive complex 2 (PRC2) subunits through bi-allelic inactivation of *SUZ12* or *EED* in a subset of MPNSTs^7–9^. However, an in-depth understanding of the molecular landscape and subtypes of MPNSTs is lacking. Here, we performed a comprehensive integrated genomic and epigenomic analysis on a cohort of 108 samples that span the entire spectrum of PNSTs with the goal to identify the drivers of malignant transformation. We discovered two distinct MPNST subgroups that we confirm and comprehensively characterized by methylation signature, copy number alterations, whole exome sequencing (WES), RNA sequencing and single-cell RNA sequencing. The transcriptional networks that define these two subgroups are sonic hedgehog (SHH) and WNT pathways. We demonstrate that targeted inhibition of SHH pathway in-vitro and in-vivo can control the growth and progression of these malignant sarcomas and establish therapeutic avenues to leverage for clinical trials.

## Results

### Methylome-based profiling identifies two distinct MPNST subgroups

**Fig. 1:**
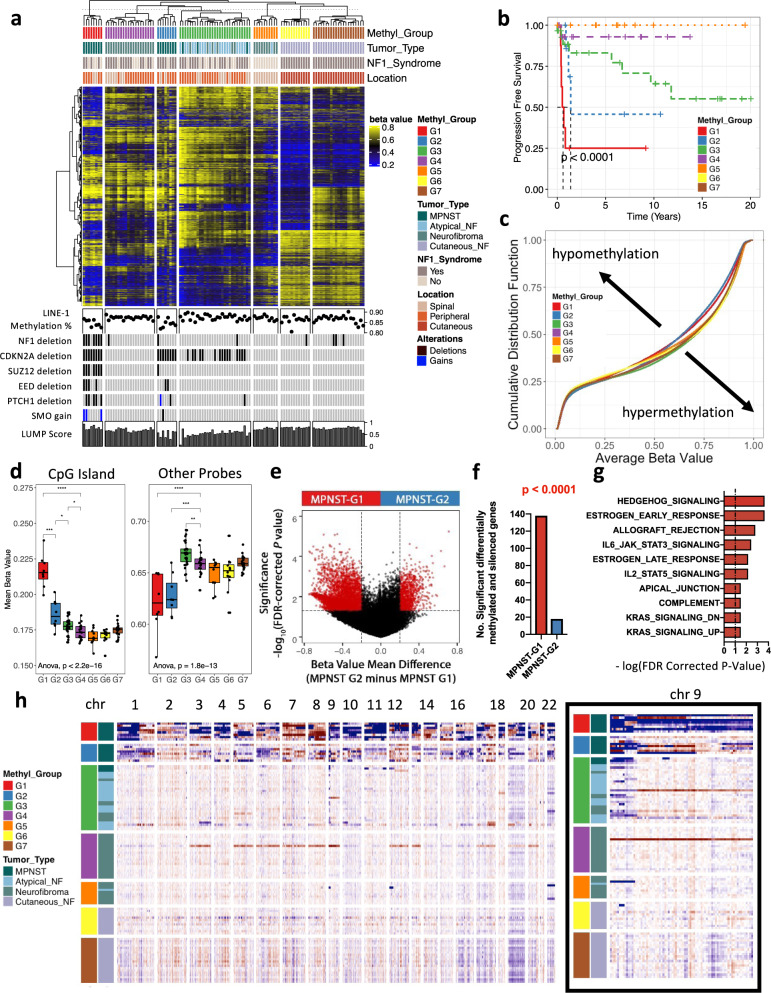
Methylation and CNV based classification of the PNSTs. **a** Unsupervised consensus hierarchical clustering of the 20,000 CpGs that show the highest median absolute deviation across the β values of 108 PNSTs. **b** Kaplan–Meier plot of progression-free survival as function of methylation subgroups. Log-Rank test (*p* < 0.0001). **c** Cumulative distribution function plot of the average β values of all CpG sites in the methylation subgroups. **d** Box plot of the mean β values of the CpG islands versus all other probes in the methylation subgroups (*n* = 108 samples). Show the median, first and third quartiles (boxes), and the whiskers encompass the 1.5X the interquartile range. One-way ANOVA (*p* < 2.2e−16 and *p* = 1.8e−13, respectively). **e** Volcano plot comparing the number of significant methylated probes of CpG islands in the promoter region between MPNST-G1 and MPNST-G2 (FDR corrected *p* value < 0.05 and mean β value difference > 0.1). **f** Differences in the number of methylated and silenced genes in MPNST-G1 versus MPNST-G2. Chi-Square Test (*p* < 0.0001). **g** Top 10 pathways affected by CpG island hypermethylation in MPNST-G1. -log (FDR corrected *P*-value) shown. **h** CNV heatmap generated from raw CONUMEE calls on methylation data for each subtype across all chromosomes. Inset shows a magnification of chromosome 9. Source data provided as source data file.

We profiled genome-wide DNA methylation patterns using the Illumina MethylationEPIC 850k array on a cohort (*n* = 108) representing the full spectrum of PNSTs (Supplementary Data 1). Unsupervised consensus hierarchical clustering of the top 20,000 most variably methylated probes yielded seven stable and robust subgroups (Fig. 1a). Cutaneous neurofibromas (33 of 33, 100%), while histopathologically indistinguishable from all other neurofibromas, resolved into homogenous methylation clusters (G6 & G7) that are distinct from all other PNSTs. Given that tumor-associated DNA methylation signatures are thought to maintain the epigenetic programs of the cell-of-origin^10,11^, the distinct methylation signature of cutaneous neurofibromas, suggests that they arise from a distinct cell-of-origin and possibly provides an explanation for the divergent clinical phenotype with lack of malignant potential for this subset of PNST neurofibromas^12^.

Atypical neurofibromas (20 of 21, 95%) and all low-grade MPNSTs (3 of 3, 100%) grouped together to form the G3 methylation cluster. G3 neurofibromas demonstrated significantly worse progression-free survival (PFS) compared to benign neurofibroma subgroups (Fig. 1b, *p* < 0.0001, log-rank test). Therefore, we propose that neurofibromas that exhibit G3 methylation signature represent a premalignant tumor at risk of malignant transformation, and this signature can be used as a potential predictive marker for malignant tendency in clinical practice. A striking finding is that the high-grade MPNSTs (*n* = 16) formed two distinct methylome clusters (MPNST-G1, *n* = 8 and MPNST-G2, *n* = 8). Based on the distinct clusters of high-grade MPNSTs (G1 and G2), we next performed consensus hierarchical clustering on the high-grade MPNST cohort alone to confirm robust delineation of two methylation subgroups (Supplementary Fig. 1a). We further validated our findings using two additional independent validation cohorts; TCGA (*n* = 5) and DKFZ (*n* = 33) datasets (Supplementary Fig. 1b–d)^10,13^. There was no difference in NF1 syndrome status, tumor size, tumor location/depth, extent of resection or adjuvant treatment (Supplementary Data 1). Most notably, survival analysis revealed that prognoses of patients with MPNST-G1 were statistically significantly worse than MPNST-G2 (median PFS of 0.6 vs 1.4 years, *p* < 0.05, log-rank test). In summary, these results reveal that there exist two distinct methylation subtypes of MPNST with very different clinical outcomes, potentially serving as a prognostic marker for MPNSTs.

### CpG island methylator phenotype in MPNST-G1

With the identification of the two distinct methylation groups MPNST-G1 and MPNST-G2, we next investigated the extent of CpG methylation in the spectrum of PNSTs. Compared to benign neurofibromas (G4), we observed an overall reduction in global DNA methylation in MPNST-G1 and MPNST-G2 (Fig. 1c, Supplementary Fig. 2a). This relative global hypomethylation phenomenon was not observed in premalignant tumors (G3) or cutaneous neurofibromas (G6&7). We then compared CpG island methylation and found that MPNST-G1 tumors have a greater number of methylated CpG islands compared to MPNST-G2 and all neurofibroma subgroups (Fig. 1d, Supplementary Fig. 2b, c). When we focused on the promoter regions, MPNST-G1 harbored more methylated CpG islands and more corresponding genes transcriptionally silenced based on gene expression data (see below) when compared to MPNST-G2 (Fig. 1e, f). Indeed, the TCGA and DKFZ cohorts confirmed that CpG islands within promoters are hypermethylated in MPNST-G1 tumors relative to MPNST-G2 tumors (Supplementary Fig. 2d, e). These results demonstrate that MPNST-G1 exhibit a CpG island hypermethylation phenotype. To determine the oncogenic programs dysregulated by the CpG island hypermethylation, we performed pathway analysis on this cohort. We found sonic hedgehog (SHH) signaling genes as the most significantly enriched pathway in MPNST-G1, which was absent in MPNST-G2 (Fig. 1g). Specifically, we found CpGs in the promoter region (cg01512589 and cg26878949) of *PTCH1*, a transmembrane protein that suppresses the SHH signaling cascade by inhibiting the oncoprotein *SMO*^14^, to be hypermethylated in MPNST-G1 but not in MPNST-G2 (Fig. 3f). This suggested that SHH pathway may be activated through epigenetic dysregulation in MPNST-G1 tumors.

### MPNST G1 and G2 harbor distinct complex copy number alterations

To investigate whether methylation sub-groups are defined by specific chromosomal aberrations, we inferred copy number profiles from the methylation data using the CONUMEE^15^ algorithm (Fig. 1h). Except for occasional small deletions affecting chromosome 17q encompassing the *NF1* locus, benign neurofibromas (G4) and cutaneous neurofibromas (G6&7) did not harbor recurrent chromosomal aberrations. In contrast, we observed the loss of chromosome 9p, encompassing the *CDKN2A* locus, in 55% of tumors in the premalignant (G3) methylation subclass. The MPNST-G1 tumors demonstrated complex copy number alterations (average 17.5 chromosomes affected), indicative of genomic instability. The most common losses affected 1p (100%), 9p (100%), 9q (87.5%), 11q (87.5%), 13q (87.5%), 17p (87.5%), 18q (75%) and 20p (75%). The most frequent gains involved 1q (50%), 8q (50%), and 19p (62.5%). In contrast, MPNST-G2 exhibited fewer chromosomal alterations (average 8.9 chromosomes affected), including loss of chromosome 1p (50%), 6p (62.5%), and 9p (62.5%), and gain of chromosome 12q (50%). In MPNST-G1, we found *PTCH1* loss (62.5%) and *SMO* gain (37.5%) in majority of samples. SHH pathway gene alterations were statistically significantly higher in MPNST-G1 compared to MPNST-G2 (75% vs 12.5%, *p* < 0.05, Fisher’s exact test).

### Mutations in PRC2 components are unique to MPNST-G1

**Fig. 2:**
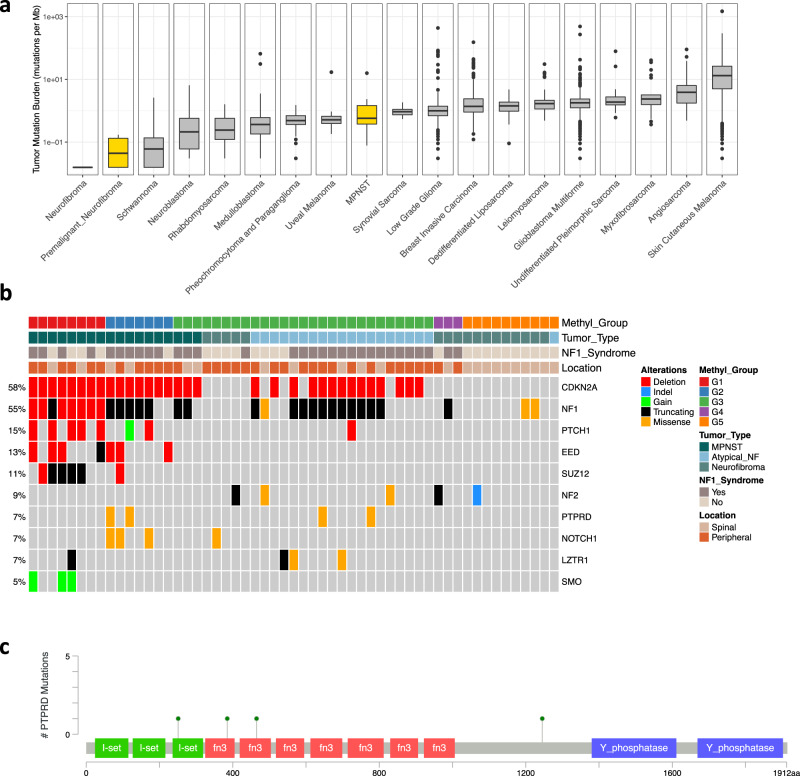
PRC2 mutations are unique to MPNST-G1. **a** Nonsynonymous mutations per megabase for benign neurofibromas, atypical neurofibromas and MPNSTs (*n* = 55 samples) compared to other childhood neuronal cancers and sarcomas from several cancer sequencing projects. Boxplots show the median, first and third quartiles (boxes), and the whiskers encompass the 1.5X the interquartile range. **b** Results from whole exome sequencing (*n* = 54) with frequency and mutations identified. **c** Schematic of the distribution of the mutations along the *PTPRD* gene.

We next performed WES to establish the mutational profile of PNSTs. The overall somatic mutation burden in this spectrum of PNSTs was low (Fig. 2a), with the mutational burden of MPNSTs (0.58 nonsynonymous SNVs per megabase) was statistically significantly higher than benign and atypical neurofibromas (0.016 and 0.049 nonsynonymous SNVs per megabase, respectively, t-test, *p* < 0.05). In keeping with previous studies, we identified *NF1* as the most frequently altered gene, with 23 of 55 samples (42%) harboring somatic mutations (Fig. 2b, Supplementary Data 3). *NF1* mutations were observed in 44% of MPNSTs, 60% of atypical neurofibromas and 18% of benign neurofibromas. When we dichotomized benign neurofibromas based on NF1 syndrome status, only 14% (2 of 14) of sporadic neurofibromas harbored somatic *NF1* gene mutations, which suggests sporadic neurofibromas may have different mechanism of RAS pathway activation. Interestingly, *NF1* mutations were significantly more prevalent in MPNST-G2 compared to MPNST-G1 (71.4% vs 12.5%, Fisher exact test, *p* < 0.05). However, we observed significantly higher rates of 17q deletions encompassing the *NF1* locus in MPNST-G1 compared to MPNST-G2 (Fig. 1a; 87.5% vs 12.5%, *p* < 0.05). We further validated these findings in the TCGA dataset, with 2 of 2 (100%) of MPNST-G1 harboring 17q deletions encompassing the *NF1* locus, while 2 of 3 (66%) MPNST-G2 tumors harboring *NF1* mutations (Supplementary Fig. 3c). Taken together, this suggests that the mechanisms of genome instability and inactivation of *NF1* is distinct between the two MPNST subgroups.

Additional recurrent but low-frequency mutations in neurofibromas include *FANCA*, *PTPRD*, *NF2*, *LZTR1*, *KNL1* and *RUNX1* (Supplementary Fig. 3a, b). In a previous study, 46% of atypical neurofibromas harbored deletions of the receptor-type tyrosine-protein phosphatase delta (*PTPRD*) genes^4^. Notably, we report a mechanism of *PTPRD* inactivation through mutations, which were private to atypical neurofibromas and MPNST-G2 tumors. We observed *PTPRD* mutations in 2 of 19 atypical neurofibromas (11%) and 2 of 7 MPNST-G2 tumors (29%). Similar to other cancers, the majority of mutations in this gene localized in the first and second fibronectin type III domains (Fig. 2c)^16,17^. *PTPRD* is a leukocyte-common antigen-related receptor tyrosine phosphatase and negatively controls the WNT/B-catenin pathway^18,19^. In addition, reduced expression of *PTPRD* was correlated with invasive status of other cancers with highly activated WNT signaling^20^. Further studies will be needed to uncover the role of *PTPRD* in MPNST-G2 tumors and cancer cell migration.

In MPNSTs, a significant proportion of recurrent mutational events converge on epigenetic mechanisms. Previous studies described nonsense mutations in *SUZ12* (24.2% of MPNSTs) and *EED* (9.8% of MPNSTs), which are core components of the PRC2 complex^7–9^. In our cohort, 22% (4 of 18) and 6% (1 of 18) of MPNSTs exhibited *SUZ12* and *EED* mutations, respectively (Fig. 2b). Most notably, the PRC2 component mutations are restricted to MPNST-G1 tumors (62.5% in MPNST-G1 vs 0% in MPNST-G2, *p* < 0.05). We confirmed this finding in the validation cohort, with 50% of MPNST-G1 (1 of 2) and none of the MPNST-G2 (0 of 3) samples harboring PRC2 component gene mutations (Supplementary Fig. 3c). Compared to other malignancies, MPNSTs have a very low tumor mutational burden and low number of recurrent mutations. Recurrent mutations in *SUZ12* and *EED* in MPNSTs further support the importance of epigenetic dysregulation in malignant transformation.

### Transcriptional profiling validates and defines two distinct subgroups of MPNSTs with SHH or WNT pathway activation

**Fig. 3:**
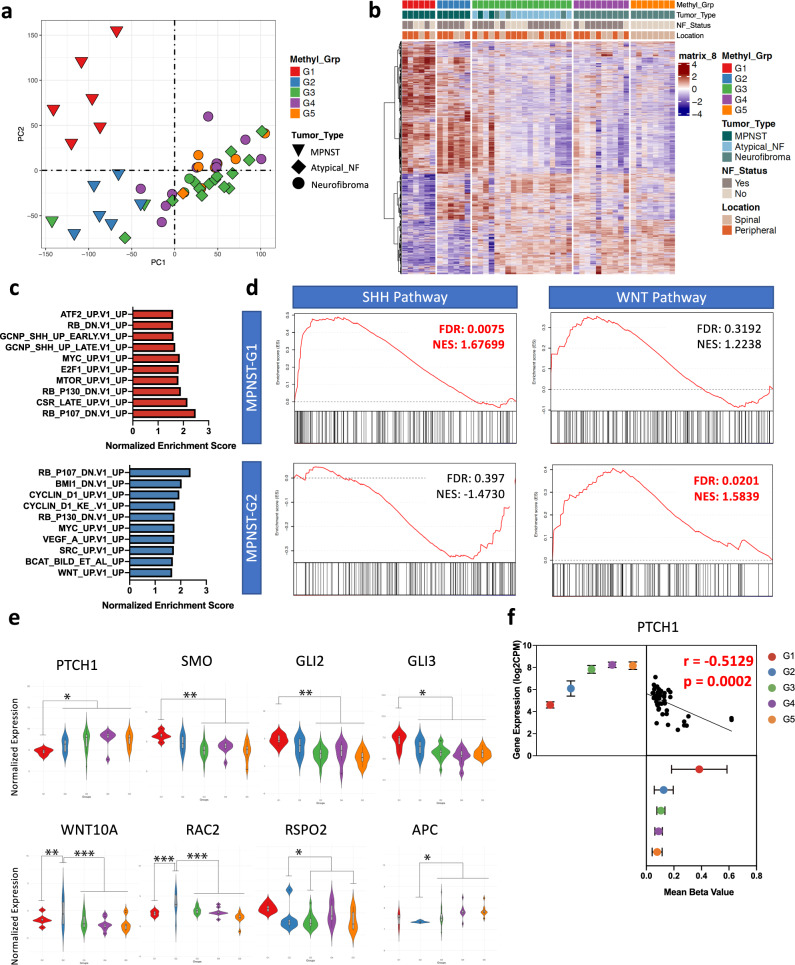
MPNST-G1 and MPNST-G2 demonstrate distinct transcriptome profiles with significant differences in critical oncogenic regulators. **a** PCA of the whole transcriptome demonstrating neurofibromas segregated from MPNSTs on PC1. MPNST-G1 and MPNST-G2 resolve into two groups on PC2. **b** Heatmap of the differentially expressed genes between the methylation subgroups (logFC > 1, FDR corrected *p*-value < 0.05). **c** Top 10 upregulated pathways in MPNST-G1 (red) and MPNST-G2 (blue) subgroups. **d** GSEA enrichment plots for the SHH and WNT pathways in MPNST-G1 and MPNST-G2. **e** Gene expression by RNA-seq of representative group of SHH and WNT pathway genes (*n* = 49 samples). Show the median, first and third quartiles (boxes), and the whiskers encompass the 1.5X the interquartile range. *p < 0.05, **p < 0.005, ***p < 0.0005 (FDR corrected *p*-value). **f** Correlation of *PTCH1* promoter hypermethylation (cg01512589 and cg26878949) with gene expression (*n* = 49 samples). Data are presented as mean values +/− SEM. Pearson’s correlation coefficient (*r* = −0.5129) and two tailed *P* values (*P* = 0.0002). Source data provided as source data file.

We next analyzed the transcriptomic profile of the two methylome-based subgroups of MPNSTs to establish whether at an expression level MPNST-G1 and MPNST-G2 maintained distinct signatures. In the principal component analysis (PCA) of the whole transcriptome, all MPNSTs clustered together and were separated from the neurofibromas by the first principal component (PC1) (Fig. 3a). Consistent with the methylation subgroupings, MPNSTs resolved into two subgroups by the second principal component (PC2). In fact, focusing analysis on MPNSTs alone faithfully reproduced the two MPNST subgroups (Supplementary Fig. 4a). Consensus clustering of the TCGA MPNST cohort also resolved into two distinct clusters that corresponded with the methylation subgroups (Supplementary Fig. 4b). Further, unbiased approaches with methylome and transcriptomic signatures agree on two distinct MPNST subgroups (Supplementary Fig. 4c; Adjusted Rand Index = 0.81, *p* < 0.001).

We next generated the differentially expressed genes between the methylation-based subgroups of all PNSTs (Fig. 3b). In Gene Set Enrichment Analysis (GSEA), we observed no difference in NF1/RAS/RAF/MEK pathway activity between MPNST-G1 and MPNST-G2 (Supplementary Fig. 5a–e). This further confirms that NF1 pathway is inactivated in both tumors despite having different mechanisms of *NF1* inactivation. Furthermore, MPNST-G1 and MPNST-G2 shared upregulation of pathways associated with *RB1* knockdown and *E2F1* upregulation, which is consistent with the frequent loss of *CDKN2A* in majority of MPNSTs (Fig. 3c). Strikingly, MPNST-G1 was enriched for PRC2 loss modules and SHH pathway activation, while MPNST-G2 was enriched for WNT/ß-catenin/cyclinD1 pathway activation (Fig. 3d, Supplementary Fig. 6).

Tumors in MPNST-G1 overexpressed genes involved in SHH signaling, such as *SMO*, *GLI2*, *GLI3*, *CCNE1* and *TGFB2* (Fig. 3e, Supplementary Data 3). In keeping with these results, we also observed a lower expression of *PTCH1* in MPNST-G1 (Fig. 3e). The pattern of CpG island promoter methylation in MPNST-G1 prompted us to investigate which genes were potentially silenced by methylation (Supplementary Fig. 7). As indicated above, there was a statistically significant *PTCH1* promoter hypermethylation in MPNST-G1 tumors compared to MPNST-G2 tumors (87.5% vs. 12.5%, *p* < 0.01, Fisher exact test). Furthermore, this inversely correlated with *PTCH1* gene expression (Fig. 3f; *r* = −0.58, *p* < 0.0001, Pearson correlation). We next used the DrugBank database to predict potential FDA approved drugs that can be repurposed for treatment using network analysis and identified smoothened inhibitors (vismodegib and sonidegib) as one of the top potential treatments for MPNST-G1 tumors (Supplementary Data 4).

In contrast, MPNST-G2 overexpressed many key WNT pathway genes, including *WNT10A*, *RAC2*, *AXIN1* and *FZD1* (Fig. 3f). This cluster of MPNSTs also significantly under-expressed *APC*, a known negative regulator of the WNT pathway and a component of the ß-catenin destruction complex^21^. In addition, *RSPO2* is a secreted ligand that potentiates WNT pathway activation, and its overexpression was previously described in a subset of MPNSTs^22^. We observed higher expression of *RSPO2* (fold change 3.7, *p* = 0.073) in MPNST-G2 samples compared to all other MPNSTs. Samples M2377 and M1933 had a 39.7-fold and 49.5-fold higher expression, respectively, of *RSPO2* compared to all other MPNSTs. Drug target analysis revealed that vorapaxar, a competitive thrombin receptor protease-activated receptor (PAR-1) antagonist, is a potential FDA approved drug candidate for treatment of MPNST-G2. PAR-1 is a positive regulator of the WNT and ß-catenin pathway (Supplementary Data 4).

### Inactivating gene fusions of PRC2 components are unique to MPNST-G1

We then examined the RNA seq data for gene fusions in all PNSTs and compared between the two MPNST subgroups. We identified an out-of-frame fusion in an atypical neurofibroma (G3) involving *CDKN2A* and *TMEM17*, which represents a mechanism for *CDKN2A* inactivation (Supplementary Data 5). MPNST-G1 harbored statistically significant more fusion events per sample compared to all other PNST clusters (Supplementary Fig. 8a–e; *p* < 0.01, ANOVA). Increased gene fusions in MPNST-G1, in addition to the previously described high copy number alterations, further lends support to chromosomal instability in this subtype. Specifically, we identified interchromosomal *JARID2-ATP5MC2* fusions in 33% of MPNST-G1 samples (Supplementary Fig. 8f–g). *JARID2* and *ATP5MC2* are located on chromosomes 6 and 12, respectively. In both cases, the fusions were predicted to be inactivating since they were classified as either out-of-frame or truncating. *JARID2* is sufficient to recruit the PRC2 to target genes, and inhibition of *JARID2* reduces PRC2 binding and loss of H3K27me3 levels on target genes^23^. *JARID2-ATP5MC2* fusions represents another mechanism by which epigenetic homeostasis is dysregulated in MPNST-G1.

### Analyzing the cellular heterogeneity of MPNSTs using single cell RNA sequencing

**Fig. 4:**
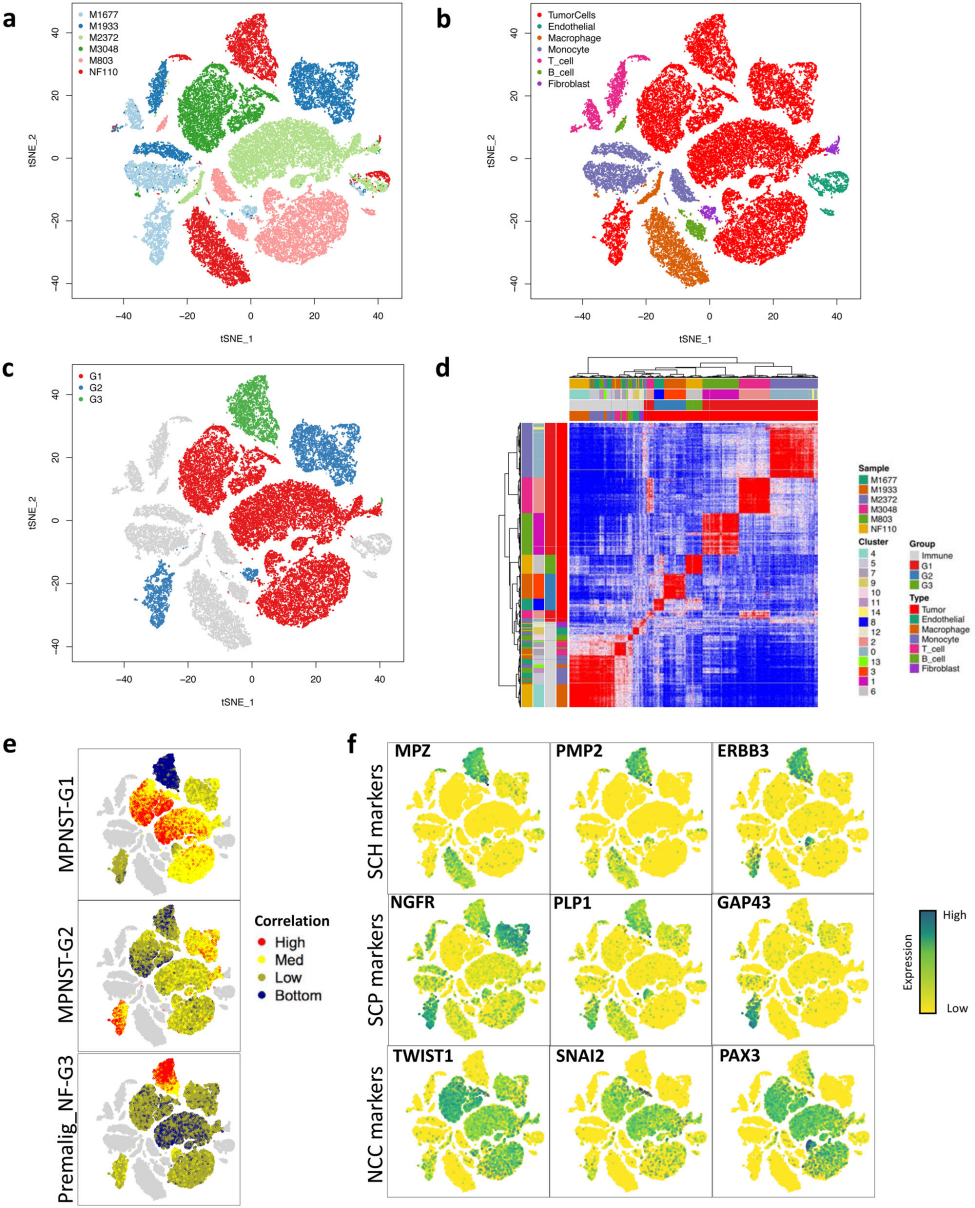
Single-nuclear RNA sequencing highlights the complex tumor microenvironment and neural crest lineage developmental hierarchy in progressing peripheral nerve sheath tumors. t-distributed stochastic neighbor embedding (t-SNE) representation of the snRNA-seq dataset. Colors represent (**a**) sample, (**b**) cell type and (**c**) methylation-based tumor subgroup. **d** Pairwise correlations between the expression profiles of single nuclei (random sample of 10,000 cells) from all 6 tumors. **e** t-SNE plots overlaid with correlation scores  to  bulk RNA signatures for MPNST-G1, MPNST-G2 and premalignant_NF-G3. **f** t-SNE plots overlaid with expression of markers for Schwann cells, Schwann-cell precursor and neural crest cells.

Cellular heterogeneity is a recognized challenging aspect of tumor biology and a well-established feature of MPNSTs^24^. We undertook droplet-based single nuclear RNA sequencing (snRNA-seq) using the 10X Genomics platform for nuclei dissociated from 6 PNSTs to infer cellular architecture. Overall, we analyzed 43,365 nuclei from 3 MPNST-G1 (M803, M3048 and M2372), 2 MPNST-G2 (M1933 and M1677), and 1 atypical neurofibroma (NF110) with a median of 2249 unique genes detected per nucleus (Fig. 4a). We estimated the identity of the cell clusters by correlating expression of genes with immune cell markers from the Human Primary Cell Atlas (HPCA) as reference (Fig. 4b)^25^. We confidently distinguished 30,518 neoplastic and 12,847 non-neoplastic cells. The transcriptional profile and copy-number profiles of neoplastic cell from each sample did not show substantial variability between cells (Fig. 4d, Supplementary Fig. 9). When we stratified based on tumor subtypes (Fig. 4c), MPNST-G2 (46.35%) and atypical neurofibromas (G3) (51.2%) had a statistically significant greater representation of immune cell populations compared to MPNST-G1 (10.6%; Supplementary Fig. 10–12a, b). The largest groups of immune cells in MPNST-G2 classified as macrophages (29.2%) and T-cells (15.4%). We further validated the difference in immune cell composition by using computational cell fraction deconvolution techniques on our bulk methylation and RNA transcriptional data from our full cohort (Supplementary Fig. 12c–e). Similarly, we observed significantly higher immune cell infiltration in premalignant neurofibroma subgroup (G3) compared to all other neurofibromas. Our data demonstrate that MPNST-G1 are predominantly neoplastic cells while immune cells constitute up to half of MPNST-G2 tumors.

### Single nuclear RNA sequencing demonstrates a neural crest cell like signature in MPNST-G1

We next analyzed the single cell data to better understand differences between the neoplastic cells of MPNST-G1 and MPNST-G2. First, we correlated the snRNA seq data of neoplastic cells of MPNST-G1 and MPNST-G2 with the bulk transcriptome signatures of all PNSTs (Fig. 4e, Supplementary Fig. 13a). The neoplastic cells from MPNST-G1 samples (M3048, M803 and M2372) had high correlation with MPNST-G1 bulk transcriptome signature and low correlation with MPNST-G2 bulk transcriptome signature. Similarly, neoplastic cells from MPNST-G2 samples (M1933 and M1677) had high correlation with MPNST-G2 bulk transcriptome signature and low correlation with MPNST-G1 signatures. Furthermore, tumoral cells in MPNST-G1 cells overexpressed *SMO*, while MPNST-G2 overexpressed *PTCH1* and *WNT11* (Supplementary Fig. 13b–d). The results further confirmed that at a cellular level MPNST-G1 and MPNST-G2 tumoral cells are distinct from each other. In addition, we inferred the neurofibroma cell composition in our full bulk RNA seq cohort, and found that MPNST-G1 tumors did not have neurofibroma cells within the tumor (Supplementary Fig. 13e). Second, to understand the cell of origin, we assessed the expression of Schwann cell lineage markers in neoplastic cells. Neurofibroma tumor nuclei remain most similar to Schwann cells, with expression of markers such as *S100B*, *PMP2*, *MPZ* and *ERBB3* (Fig. 4f, Supplementary Fig. 14, Supplementary Table 7). In contrast, majority of MPNST neoplastic cells had lost these Schwann cell markers, suggesting that these cells have dedifferentiated into a more primitive cell state. Intriguingly, we observed overexpression of factors known to play canonical roles in the early neural crest cell specification (*TWIST1*, *SOX9, SNAI2, OTX2, PAX3* and *PAX6*)^26,27^ in MPNST-G1 tumor cells. Neural crest cells are multipotent developmental cell population characterized by molecular diversity, which can give rise to Schwann cells, neurons, melanocytes, smooth muscles, cartilage and bone^28^. MPNST-G2 neoplastic cells resembled more of a Schwann-cell precursor-like cell phenotype by overexpressing *GAP43*, *PLP1* and *NGFR*, with an absence of Schwann cell markers (*S100B, PMP2, ERBB3, MPZ*)^29^. Finally, we performed trajectory analysis on the tumoral cells and demonstrated a pseudotemporal continuum from atypical neurofibroma (Schwann cell) to MPNST-G2 (Schwann cell precursor cell) to MPNST-G1 (neural crest cell), which further supports that these tumors fall along the developmental trajectories of neural crest lineage (Supplementary Fig. 12e, f).

### SHH pathway activation drives malignant transformation and provides an effective therapeutic target

**Fig. 5:**
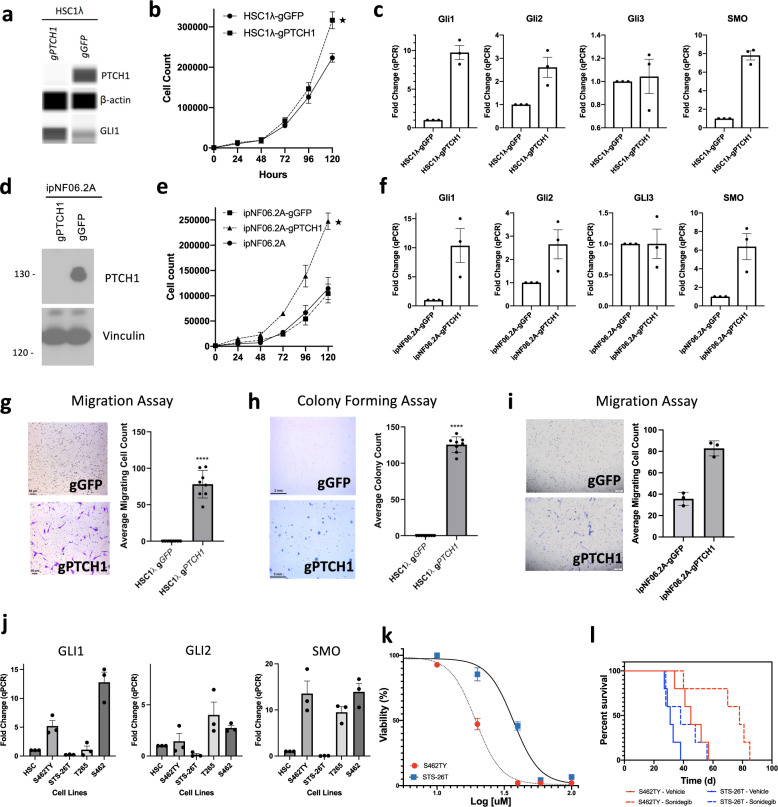
SHH pathway is important for malignant transformation in a subset of MPNSTs and a therapeutic target. **a** Capillary-based immunoassay (WES) confirming knockout of PTCH1 and subsequent increased GLI1 activity in HSC1-λ cells. **b** Trypan blue counts of parental HSC1-λ and PTCH1-knockout clone. Error bars, s.e.m.; *n* = 3 biologically independent experiments. **c** PTCH1-knockout in HSC1-λ cell lines leads to upregulation of SHH pathway (GLI1, GLI2 and SMO). Error bars, s.e.m.; *n* = 3 biologically independent experiments. **d** Protein blot confirming knockout of PTCH1 in ipNF06.2A cells. **e** Trypan blue counts of a parental ipNF06.2A cells and PTCH1-knockout clone. Error bars, s.e.m; *n* = 3 biologically independent experiments. **f** PTCH1-knockout in ipNF06.2A leads to upregulation of SHH pathway (GLI1, GLI2 and SMO). Error bars, s.e.m; *n* = 3 biologically independent experiments. **g** Representative images and quantitative cell migration in HSC1-λ cell lines. Error bars, s.e.m; *n* = 8 biologically independent experiments. **h** Representative images and quantitative colony formation in HSC1-λ cell lines. Error bars, s.e.m; *n* = 8 biologically independent experiments. **i** Representative images and quantitative colony formation in ipNF06.2A cells. Error bars, s.e.m; *n* = 3 biologically independent experiments. **j** 4 MPNST cells lines were screened for SHH pathway activation. Error bars, s.e.m; *n* = 3 biologically independent experiments. **k** IC50 curves of S462TY and STS-26T cells treated with sonidegib. Error bars, s.e.m; *n* = 3 biologically independent experiments. **l** Survival curves of mouse xenografts of S462TY and STS-26T treated with sonidegib at a dose of 20 mg/kg/day or vehicle starting day 14. Median survival for S462TY-Sonidegib vs S462TY-Vehicle (78 vs 45 days, log-rank test, *p* = 0.03). Source data provided as source data file.

The accumulation of methylation, mutational and transcriptomic data have all supported that there are two distinct MPNST subgroups formed through two distinct pathways; SHH and WNT. In MPNST-G1, multiple biological mechanisms appear to converge on the SHH pathway playing a central role in malignant progression. We therefore hypothesized that SHH activation in immortalized human Schwann cells (HSC1λ) may be sufficient to induce transformation and aggressive phenotype. To activate the SHH pathway, we generated *PTCH1*-knockout HSC1λ (HSC1λ-g*PTCH1*) cell lines (Fig. 5a). The loss of *PTCH1* resulted in increased expression of *SMO*, *GLI1* and *GLI2* in the HSC1λ cell lines, which are SHH pathway genes (Fig. 5c). Furthermore, *PTCH1*-knockout resulted in statistically significant increase in cellular proliferation, anchorage independent growth and migration (Fig. 5b, g, h). To further validate these findings, we also knocked out *PTCH1* in an immortalized neurofibroma cell line (ipNF06.2A)^30^. Again, *PTCH1*-knockout in ipNF06.2A cells induced activation of the SHH pathway and a significant increase in cellular proliferation and migration (Fig. 5d–f,i). We next tested *PTCH1*-knockout cells in vivo by injection into the flanks of immunodeficient mice. In 75% of mice HSC1λ-g*PTCH1* formed tumors in the flank, compared to 0% of mice injected with control parental HSC1λ-gGFP cells. Since *NF1* is frequently co-deleted in the spectrum of PNSTs, we also engineered cells to knock out both *NF1* and PTCH, creating HSC1λ *NF1*^−/−^; g*PTCH1* cells. These cells grew as xenograft tumors in all mice (100%), while the *NF1*^−/−^ HSC1λ cells (with *PTCH1* intact) formed tumors in only 50% of mice (Supplementary Fig. 14). These results validate that SHH pathway induces malignant phenotype in vitro and in vivo.

We next characterized a panel of established human MPNST cell lines (S462-TY, T265, S462, and STS-26T) with respect to the status of activation of SHH and WNT pathway. We identified significantly elevated levels of *GLI1*, *GLI2*, and *SMO* in 3 of 4 MPNST cell lines (S462, S462TY, and T265) that interestingly also have PRC2 alterations (Fig. 5j). Similarly, STS-26T with WT PRC2 complex demonstrated significantly higher levels of *CCND1* expression (Supplementary Fig. 15a). To confirm that the MPNST cell lines were consistent with our established MPNST subgroups, we assessed the methylation profiles. As expected, cell lines S462, S462TY and T265 clustered with MPSNT-G1 tumors, while STS-26T clustered with MPNST-G2 tumors (Supplementary Fig. 15b). These results validate our tumor-based genomic analysis. Given that our genomic data identified the upregulation of the SHH pathway to be a driver of MPNST-G1 tumors and our transcriptomic network analysis identified SMO inhibitors as a potential drug candidate, we hypothesized that sonidegib (SMO inhibitor) could provide a therapeutic benefit to MPNST lines with SHH pathway activation while not impacting the other cell line. To test this hypothesis, we treated S462TY (MPNST-G1; SHH activated) and STS-26T (MPNST-G2; WNT activated) with sonidegib for 48 h at varying doses (Supplementary Fig. 16b, c). In vitro treatment of S462TY with sonidegib resulted in a twofold lower half-maximal inhibitory concentration (IC50) compared to STS-26T (Fig. 5k). To validate this finding in vivo, we injected S46TY and STS-26T cells into the flank of NOD-SCID mice and treated with sonidegib (20 mg/kg/day) until end point (tumor size >1.5 cm). S462TY xenografts treated with sonidegib survived statistically significantly longer than xenografts treated with vehicle (median survival 78 vs 45 days, log-rank test, *p* = 0.03, Fig. 5l). In contrast, STS-26T xenografts treated with sonidegib did not have improved survival consistent with the lack of SHH pathway activation in this cell line. These results lend additional validation of the genomic/epigenomic findings, as well as provide the preclinical evidence to support clinical trials using SMO inhibitor in MPNST-G1 subgroup.

## Discussion

**Fig. 6:**
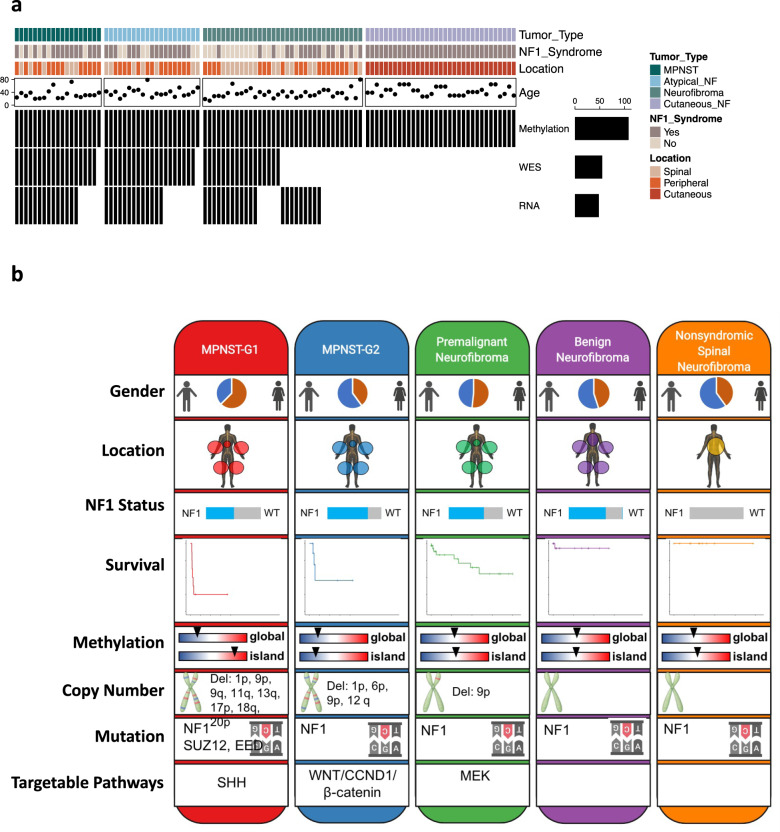
Synopsis of PNST subgroups. Overview of the PNST molecular subgroups and their demographic, genetic and epigenetic characteristics. **a** Summary of the demographic data and the available molecular datasets for cohort of PNSTs. **b** A schematic representation that summarizes the major molecular findings and conclusions of our study. Created with Biorender.com.

There is a considerable unmet need to identify clinically relevant and actionable therapeutic targets for MPNSTS, as present treatment options ar limited. Furthermore, identifying predictive markers of malignant transformation would be most impactful to care of patients with sporadic or NF-1 associated PNSTs. Our in depth comprehensive integrative genomic and epigenomic analysis of the spectrum of PNSTs identifies two molecularly distinct pathways that can drive the growth and progression of MPNSTs: MPNST-G1 and MPNST-G2, each have a distinct outcome with MPNST-G2 demonstrating more than double the RFS compared MPNSTs-G1. Our study highlights the importance of future studies with larger cohorts of tumors to better understand the molecular alterations that drive malignant transformation and the clinical significance of the two MPNST subgroups. Most importantly the two distinct subgroups can unmask molecular rationale to explore therapeutic vulnerabilities in clinical trials (Fig. 6).

Data from this study support that epigenetic dysregulation is a defining feature of MPNSTs. Although MPNSTs harbor very few recurrent somatic genetic events, many of the recurrent genetic events converge on genes important in post-translational modification of histones, specifically H3K27me3. Alterations in the PRC2 components, through mutations and gene fusions, are uniquely restricted to MPNST-G1. PRC2 mainly mediates transcriptional repression and has essential roles in maintenance of cell identity and differentiation. In embryonic stem cells, the loss of PRC2 induces hypermethylation of CpG islands in the promoter regions near key developmental genes^31,32^. PRC2 deficient MPNST-G1 demonstrated a CpG island promoter hypermethylation phenotype. In nerve injury models, epigenetic reprogramming of a Schwann cell is induced after nerve injury through inhibition of PRC2 and subsequent activation of the SHH pathway^33^. We propose that MPNST-G1 tumors hijack this nerve regeneration machinery through inactivating molecular alterations of MPNST-G1 that result in dysregulation of the SHH signaling cascade. In contrast, PRC2 dysregulation is not evident in MPNST-G2 though this subgroup demonstrates global hypomethylation. The mechanism for MPNST-G2 hypomethylation needs further future investigation.

Notably, we found that SMO inhibitors are a potential therapeutic option for treatment of SHH-MPNST subgroup of tumors. MPNST-G1 tumors were enriched for deletions of *PTCH1*, a negative regulator of the SHH pathway, and gains of *SMO*, a positive regulator of the SHH pathway. In addition, we demonstrate promoter hypermethylation of *PTCH1* and a corresponding repression of gene expression. Dysregulation and activation of the SHH signaling cascade is implicated in several cancers, but to date not studied in detail in PNSTs^34^. Transcriptome profiling further highlighted SHH pathway activation in MPNST-G1, with computational network analysis-based drug screen suggesting the SMO inhibitors are one of the top drug classes for treatment of MPNST-G1. Our in-vitro and in-vivo data also demonstrated that SHH pathway activation is sufficient to drive malignant phenotype in Schwann cell and neurofibroma cell lines. Most importantly, SMO inhibitors demonstrated efficacy in treating MPNST cell lines that had SHH pathway activation. Taken together, our data provides evidence to support a clinical trial for SMO inhibitors in the MPNST-G1 subgroup of tumors.

We have demonstrated that the second molecular subgroup of MPNSTs (MPNST-G2) have significant activation of the WNT/ß-catenin/cyclinD1 pathways. Consistent with our data, a forward genetic screen utilizing the *Sleeping Beauty (SB)* transposon based somatic mutagenesis system identified many cooperating mutations affecting WNT signaling regulators in mice MPNST-like tumors^35^. Similarly, WNT pathway activation induces malignant behavior in human Schwann cells and are required for tumor maintenance in MPNST cells^22,36^. Our data demonstrates that activation of the WNT pathway through knockout of APC is sufficient to drive malignant transformation in Schwann cell lines. In addition, we demonstrate that MPNST-G2 harbor significantly larger population of macrophages within the tumor microenvironment, which may be a potential therapeutic target. We definitively show that MPNST-G2 tumors are different biologically from MPNST-G1 and have unique set of targetable oncogenic pathways driving tumorigenesis and moreover distinct clinical outcomes.

At a single cell level, the tumor cells in each tumor demonstrated high correlation with the bulk transcriptomic signatures of their respective MPNST subgroup. Most interestingly, the malignant cells from MPNST-G1 and MPNST-G2 resembled various stages of the Schwann cell lineage. MPNST-G1 tumor cells resemble a neural crest cell-like state, while MPNST-G2 tumors resembled a Schwann cell precursor-like state, which is an intermediate cell type between neural crest cells and Schwann cells. Hedgehog signaling is essential for the survival of neural crest cells, and blocking this cascade has shown to decrease cell proliferation and induce apoptosis^37–39^. Collectively, the data strongly supports the existence of two distinct cells of origin for the subtypes of MPNST.

In conclusion, integrated multi-platform genomic and epigenomic analysis shows that there are two distinct molecular subgroups of MPNSTs: MPNST-G1 and MPNST-G2 that can inform on predictive biomarkers for prognosis, leverage therapeutic vulnerabilities and drivers of malignant transformation. We found distinct mechanisms that drive MPNST formation and have shown through in vitro and in vivo preclinical models that targeting these oncogenic programs can be leveraged towards therapeutic options.

## Methods

### Patient samples and clinical annotation

Tumor samples and peripheral blood of patients were collected from the University Health Network Brain Tumor Bank (Toronto) and the Mount Sinai Hospital Sarcoma Tumor Bank (Toronto, Canada) under institutional review board (IRB)-approved protocols with patient informed consent. Samples were collected fresh from the patients at the time of surgical resection and stored at −80 °C. Additional samples were obtained from the Children’s Tumor Foundation (CTF, New York, USA). In summary, we used 108 samples in this study (19 MPNSTs, 22 premalignant neurofibromas, 34 plexiform neurofibromas and 33 cutaneous neurofibromas). Pathologic diagnosis was confirmed by at least two experienced pathologists using diagnostic formalin-fixed and paraffin-embedded blocks to confirm diagnosis of peripheral nerve sheath tumors and to subtype tumors according to recognized histopathological classifications. Given the tendency for local recurrence and distant metastasis in MPNSTs, PFS was used as the primary outcome of interest in this study. PFS was defined as local tumor growth after gross total resection, tumor progression following subtotal resection, or distant metastasis. Time to recurrence was determined by calculating the elapsed time from the date of index surgery to first postoperative imaging documenting tumor recurrence.

### DNA and RNA processing

DNA was extracted from fresh-frozen tumor tissue and normal tissue using the DNeasy Blood and Tissue Kit (Qiagen, USA). Total RNA was isolated from tumor using the RNeasy Mini Kit (Qiagen, USA). DNA and RNA were quantified using Nanodrop 1000 instrument (Thermo Scientific, USA) and integrity assessed by either agarose gel electrophoresis (DNA) or Agilent 2100 Bioanalyzer (RNA; Agilent, USA) at the Princess Margaret Cancer Genomics Centre (PMGC, Toronto, Canada). DNA (0.5-1μg) was used for bisulfite treatment (Zymo Research, Irvine, USA).

### Genome-wide DNA methylation analysis

Methylation profiling was performed on bisulfite-treated DNA using the Illumina Infinium MethylationEPIC BeadChip array (Illumina, San Diego, USA) at the PMGC (Toronto, Canada). Generated raw methylation files were imported and preprocessed with the statistical programming language R using the minfi package (Bioconductor). Data was normalized and failed probes with a detection *P*-value threshold > 0.01 were removed from further downstream analysis. Further filtering of the data was performed, as previously described, by removing all probes that fit one of these criteria: (1) probes that overlapped with known single nucleotide polymorphisms, (2) probes located on X and Y chromosomes, or (3) cross-reactive probes. Unsupervised consensus hierarchical clustering was performed with ConsensusClusterPlus package (Bioconductor) on beta values using Euclidean distance and Ward’s linkage method with 1000 resampling steps (epsilon = 0.8). We selected the top 20,000 probes that showed the highest median absolute deviation (MAD) across the ß-values for clustering. The unscaled methylation levels were shown in a heatmap from unmethylated state (purple color) to methylated state (red color). Differentially methylated probes were identified using *limma* based modeling approach (Bioconductor) for the methylation subclasses. Absolute mean β-value difference > 0.1 and adjusted *p*-value (FDR-corrected) < 0.05 were considered to be significant.

### Whole exome sequencing

Whole-exome sequencing of 54 PNSTs and 20 matched blood samples was performed by The Centre for Applied Genomics (TCAG, Toronto). Genomic DNA libraries were prepared using Agilent SureSelect Human Exome Library Preparation V5 kit with the Agilent Bravo Automation System and paired end sequencing on a HiSeq 2500 platform to a median of 60X. In brief, read pairs were aligned to the hg19 reference genome using BWA-MEM v 0.7.12^40^ with default parameters. PCR duplicate marking, indel realignment and base quality score recalibration were performed using Picard v1.72 and GATK v3.6.0^41^. We obtained germline variant calls by joint genotyping and variant quality score recalibration on 20 peripheral blood controls, in adherence to the GATK Best Practices framework^42^. Data quality assessment was performed using Picard tool CalculateHSMetrics. We identified somatic mutations using Mutect V1.1.6^43^ and Strelka v1.0.13^44^ for 20 tumors with matched peripheral blood controls and Mutect2 V1.1^43^ for 35 unmatched tumor samples. Variants with allele fractions <10% were removed to control for potential sequencing artifacts. Since we lacked normal control for a subset of samples, putative somatic variants were retained by filtering likely germline variants with GnomAD^45^ population frequency >0.01% (r2.0.1). TGL frequency database of variants of <1% were retained to filter out initial passenger events. We annotated variants using Variant Effect Predictor v.92.0^46^, OncoKB Precision Oncology Knowledge Base^47^, CancerHotspots.org^48^ and dbNSFP database^49^. Tumor mutational burden is calculated as the fraction of total number of nonsynonymous (protein altering) somatic mutations across the whole exome capture space in Mb.

### RNA sequencing and analysis

The isolated RNA was processed using the Illumina TruSeq Stranded Total RNA kit with Ribo-Zero Gold Prep kit according to manufacturer’s protocol. Libraries were sequenced on the Illumina NextSeq500 platform with 75-cycle paired-end protocol to obtain minimum 40 million reads. Sequence data was processed and aligned to human reference genome (hg38) using STAR aligner (v.2.6.0)^50^. Next, duplicate reads were removed. Reads were sorted using SamTools (v.1.3)^51^. Gene expression in raw counts for each sample was calculated by the algorithm “featureCounts” in the package Rsubread (v.1.5.0). Data was normalized by counts-per-million (CPM) and then subjected to trimmed means of M (TMM) using edgeR^52^ (v.3.22.3). CPM cutoff values were determined empirically by identifying the minimum value required to achieve the best normalization across samples. Differentially expressed genes (DEG) was determined using Quasi-likelihood F-test in EdgeR^52^. Pathway analysis was performed on the DEG from the indicated pairwise analysis using Gene Set Enrichment Analysis (GSEA) package (Broad Institute).

### Computational FDA drug mapping

In order to discover potential therapeutic agents, we used the Enrichment Map App in Cytoscape to perform post-analysis with FDA approved drug targets. A scoring system was formulated to first select drugs by the number of target genes in the leading edge of significant GSEA pathways for indicated comparison. Then each drug was ranked by the number of pathways targeted. Finally, the number of significant genes targeted were divided by the total number of target genes of the drug to assess the specificity. This scoring system selected the drugs targeting the greatest number of driving genes in significant biological pathways with high specificity. The resulting list of drugs were grouped by common targets to produce a high-level summary of the class of drugs with the highest possibility of effective treatment.

### Fusion calls

Fusion genes for cohort of PNSTs were identified using FusionCatcher v1.1.0^53,54^ with default parameters, which aligns reads to the human reference genome (GRCh38) using Bowtie^55^ (v1.2), Bowtie2^56^ (v2.3), Star^57^ (v2.7) and BLAT^58^ (v0.35). Adjacent and read-through fusions were filtered out from analyses. To reduce false positive detection of genes with similar sequence homology, gene fusions with Counts_of_common_mapping_reads = 0 were selected.

### Droplet-based single nuclear RNA sequencing and analysis

Flash frozen archived tumor specimens were minced with sterile scalpel and mechanically dissociated with a dounce tissue grinder (size A and B, Sigma Aldrich) in ice cold lysis buffer (0.32 M sucrose, 5 mM CaCl2, 3 mM MgAc2, 0.1 mM EDTA, 20 mM Tris-HCl, 40U/ml RNase inhibitor and 0.1% Triton X-100 in DEPC-treated water). Homogenized tissues was centrifuged at 800 × *g* for 10 min at 4 °C and resuspended in 1 m of wash buffer (1× PBS, 1% BSA and 0.2 U/μl RNase Inhibitor). Nuclei were filtered twice in 40 μm strainers (Flowmi cell strainer, Sigma Aldrich). Isolated nuclei were stained with DAPI at concentrations suggested by the manufacturer and sorted by FACS (BD Influx BRV, Becton Dickinson Biosciences). Nuclei were collected, washed and resuspended in wash buffer. Nuclei were counted and appropriate volume for each sample was calculated for a target capture of 6000 nuclei. Samples were loaded onto a 10× Chromium controller using the Chromium Single Cell 3’ Library & Gel Bead Kit v3 (10× Genomics). After droplet generation, Libraries were sequenced on an Illumina NovaSeq (10x specific protocol) with >50, 000 reads per cell.

CellRanger (10× Genomics) analysis pipeline was used for library demultiplexing, read alignment to human genome GRCh38 and UMI quantification per gene per cell. High-quality cells with >1500 unique genes detected and <1.5% reads attributed to mitochondrial transcripts were retained. Genes detected in less than three cells were removed. The raw gene expression matrix was normalized, and variance stabilized by SCTransform in Suerat version 3.0 using UMI count and percent reads aligned to mitochondrial transcripts as covariates (Stuart et al. 2018). Principal component analysis was performed using highly variable genes (FDR < 0.001) identified by scran and the number of significant principal components (PC,10) were determined based on the inflection point of the “scree” plot. Principle components were corrected for batch effect using Harmony (v.01)^59^. The first 10 PC spaces were used to build a shared-nearest-neighbor graph. Clusters were identified by optimizing the modularity function within its space with a resolution set to 0.1 and clustering results were visualized using t-SNE of the selected principal components.

### scRNA-seq pseudotime analysis

Pseudotime analysis was performed as previously described^60^ using Monocle2^61^. Briefly, raw UMI counts from the cleaned and processed Seurat objects was used to generate CellDataSet objects by normalizing the data using a negative binomial distribution with fixed variance. The CellDataSet object was processed to estimate sequencing depth (estimateSizeFactors), gene dispersions (estimateDispersions) and per-cell coverage. The data was further filtered to keep high-quality cells with >500 genes. In addition, genes were filtered to retain genes present in greater than 10 cells. The DDRTree algorithm included with Monocle2 was used to reduce the dataset two dimensions and the marker genes that differentiated the clusters were used to guide the trajectory inference. Relative pseudotime was generated through a linear transformation relative to the cells with the lowest and highest pseudotimes.

### Cell type classification

Cells were assigned to different cell types based on a consensus of (1) similarity of expression profiles, (2) copy number profiles and (3) expression of canonical markers. First, unsupervised hierarchical Pearson clustering with Ward linkages on the matrix of correlation was performed to correlate the expression profile of each cell to every other cell. Two major clusters of putative neoplastic and non-neoplastic cells were identified. Second, CNAs of neoplastic and non-neoplastic cells were inferred from the snRNA-seq data using inferCNV (v.1.1.1)^62^. After the genes were ordered according to the human GRCh38 assembly, a heatmap illustrating relative expression intensities of neoplastic nuclei to reference population across the genome was generated for visualization. Almost all neoplastic clusters harbored multiple CNAs throughout the genome, while the non-neoplastic cells were generally lacking CNAs. Finally, FindAllMarkers in Seurat was used to identify differentially expressed genes in each cluster, which were then inspected for canonical immune and stromal cell markers.

### Cell viability assay

Immortalized human Schwann cell (HSC1λ) and immortalized neurofibroma cell (ipNF06.2A^30^) were obtained from Dr. Margaret Wallace’s laboratory at the University of Florida. MPNST cell lines (STS-26T^63^, S462^64^, S462TY, T265^65^) were obtained from Dr. David Largaespada’s laboratory at the University of Minnesota. All cell lines were cultured with Dulbecco’s modified Eagle’s medium (DMEM, Wisent Technologies) supplemented with 10% fetal bovine serum (FBS, Wisent Technologies) and 1% penicillin/streptomycin. Cells were grown at 37 °C and 5% CO_2_. For direct cell counting, 1 × 10^5^ cells were plated in triplicates into 6 well plates in 2 ml of medium. After incubation times (days 1–5), cells were collected and analyzed for cell count and cell viability. Cells were directly counted using Trypan blue and the Beckman Coulter Vi-CELL (12-sample carousel) Cell Viability Analyzer (Beckman Coulter). IC50 assays were performed in 96 wells by seeding 5000 cells in triplicate overnight. Cells were treated with sonidegib (NVP-LDE225, Selleck Chemicals) the following day with increasing drug concentrations and read by CellTitre-Glo luminescent cell viability assay in accordance with the manufacturer’s instructions (Promega, G7570) on a 96-well plate reader (GloMax-96 microplate luminometer; Promega).

### CRISPR/Cas9 knockout cell line preparation

Guide RNA oligo sequences were generated using CRISPR-Cas9 Design Tool (www.crispr.mit.edu/). The guide RNA sequences chosen had the least number of potential off-target sites predicted by the CRISPR-Cas9 Design Tool. To generate CRISPR/Cas9-modified ipNF06.2A cell lines, sequences were subcloned into pX330: gGFP;5’-GGATACTTCTTCGAACGTTT, gPTCH1;5’-TGCTTTTAATCCCACCGCGA. Cells were co-transfected with one pX330 construct and pRNAT-H1.3 (Hygro) control plasmid expressing GFP and hygromycin resistance genes. ipNF06.2A-gPTCH1 and ipNF06.2A-gCTRL cells were selected with hygromycin and monoclonal lines were screened. CRISPR/Cas9-modified HSC1λ cell lines were generated using lentiviral transduction of *Cas9* and a guide RNA: gGFP; 5’- CACCGAGCTGGACGGCGACGTAAAG, gPTCH1;5’- GCCTATGGCGCGGCAGACCACCCAC, gAPC;5’CACCGAACAGCATCGAGCCAACCTCACCGCCGAGCAGCGGCTAGGCTTCCACCGAAGCCTAGCCGCTGCTCGGCACCGCCGGAAGCCTAGCCGCTGCT. Lentiviral particles were produced in 293T cells with viral packaging plasmids. After 24 h, the viral supernatant was collected and added to adherent HSC1λ cells, supplemented with 6 μg/ml polybrene. After lentiviral transduction, cells with the correct construct were selected with 2 μg/ml puromycin. Genotype was confirmed with PCR and sequencing. These cells were grown in media with puromycin supplemented through all downstream applications.

### Western blot

Traditional western blots were performed using standard protocols. Primary antibodies for B-Actin(1:1000, Cat #8457S, Cell Signaling Technologies), Vinculin(1:30000, Cat #V9264, Sigma Aldrich), PTCH1(1:500,Cat#MAB41051, R&D systems) and APC(1:500, Cat #15270, Abcam) were used. Knock out HSC1λ cell line genotypes were confirmed with capillary electrophoresis-western blot using a WES capillary electrophoresis device (ProteinSimple, San Jose, CA, USA) according to the manufacturer’s instructions. Primary antibodies against B-Actin (Cell Signaling Technologies #8457S, 1:10), PTCH1 (Cell Signaling Technologies #2468, 1:10), GLI1 (Cell Signaling Technologies #3538, 1:10).

### Anchorage independent growth assay

6-well plates (Corning) were prepared with bottom agar (3.2%) and top agar (0.48%) composed of low melting point agarose in DMEM full media. The bottom agar was allowed to solidify before 10,000 cells in top agar were plated and allowed to solidify. DMEM media supplemented with 10% FBS and 2 ug/ml puromycin was plated over the cells in the 6-well plates and incubated under standard conditions. After 14 days, top media were removed, and cells were fixed in 10% formalin (Fisher Scientific) containing 0.0005% crystal violet (Sigma) for 1 h at room temperature. Colonies were imaged on Leica S8 AP0 microscope with 12 images per cell line. Automated colony counts were done using ImageJ software^66^ using TKS Batch Count Colonies macro (courtesy of StarrLab). Results shown are a representative example of at least 3 independent experiments.

### Transwell migration assay

Cell migration assays were performed using Transwell inserts with 8 μm pore size polycarbonate membranes (Corning). Briefly, cells were resuspended in serum free media and 1000 cells were seeded into inserts. The lower chamber for each well was set up with 500 μl of media supplemented with 10% FBS as a chemoattractant. After 24 h, the cells on upper membrane surface were removed mechanically. The membranes were then fixed with 0.1% crystal violet for 15 min and then mounted on slides. The number of migrating cells were observed with brightfield microscope, and manually counted.

### Xenograft models

All animal procedures were carried out according to animal use protocols approved by the Institutional Animal Care Committee. Immunodeficient NOD-*Rag1*^*null*^ *IL2rg*^*null*^ mice (The Jackson Laboratory) received approximately 3 million cells via subcutaneous flank injection. Cells were in media and Matrigel in a 1:1 ratio. Mice were injected with HSC1λ gGFP (*n* = 4), HSC1λ gPTCH1 (*n* = 4), HSC1λ NF1^−/−^ gPTCH1 (*n* = 4), HSC1λ NF1^−/−^ (*n* = 12), HSC1λ gGFP (*n* = 4), HSC1λ gAPC (*n* = 4). Mice were monitored daily. Mice were sacrificed 4 months post-injection and tumors were harvested. For the sonidegib drug treatment, non-obese diabetic severe combined immune deficiency spontaneous male mice (NOD-SCID-Prkdc^scid^) received 5 × 10^6^ cells via subcutaneous flank injection. Mice were randomly selected to receive S462TY (10 mice) or STS-26T cells (10 mice). Cells were in a 1:1 media and Matrigel suspension. On day 14, mice were treated with sonidegib (Sellekchem) dissolved in vehicle (PEG 400/5% dextrose in water), at a dose of 20 mg/kg/day, or with vehicle alone. Mice were monitored daily, and tumor size was measured with calipers. Mice were sacrificed when they reached end point (tumor size >1.5 cm in any one dimension).

### Reporting summary

Further information on research design is available in the Nature Portfolio Reporting Summary linked to this article.

## Supplementary information

Supplementary Information

Reporting Summary

Description of Additional Supplementary Informaation

Supplementary Data 1

Supplementary Data 2

Supplementary Data 3

Supplementary Data 4

Supplementary Data 5

Supplementary Figure Legends

SourceData

## Data Availability

Raw sequencing data for all datatypes have been deposited into public repositories and publicly available. Methylation (idat) datasets has been deposited to GSE207207 [https://www.ncbi.nlm.nih.gov/bioproject/PRJNA854154], bulk RNA (fastq) datasets has been deposited to GSE207399 [https://www.ncbi.nlm.nih.gov/bioproject/PRJNA855245], snRNA(fastq) has been deposited to GSE207400 [https://www.ncbi.nlm.nih.gov/bioproject/PRJNA855244], and WES datasets has been deposited to PRJNA854920. Source data are provided as Source Data file.

## Code availability

No special code was used in this study, and code for all figures in the study are available for research purposes from the corresponding authors on request.
