# Supplementary material for: Multiplatform molecular profiling uncovers two subgroups of malignant peripheral nerve sheath tumors with distinct therapeutic vulnerabilities
Source: Nat Commun. 2023 May 10;14:2696. doi: 10.1038/s41467-023-38432-6 (PMC10172395; doi:10.1038/s41467-023-38432-6)

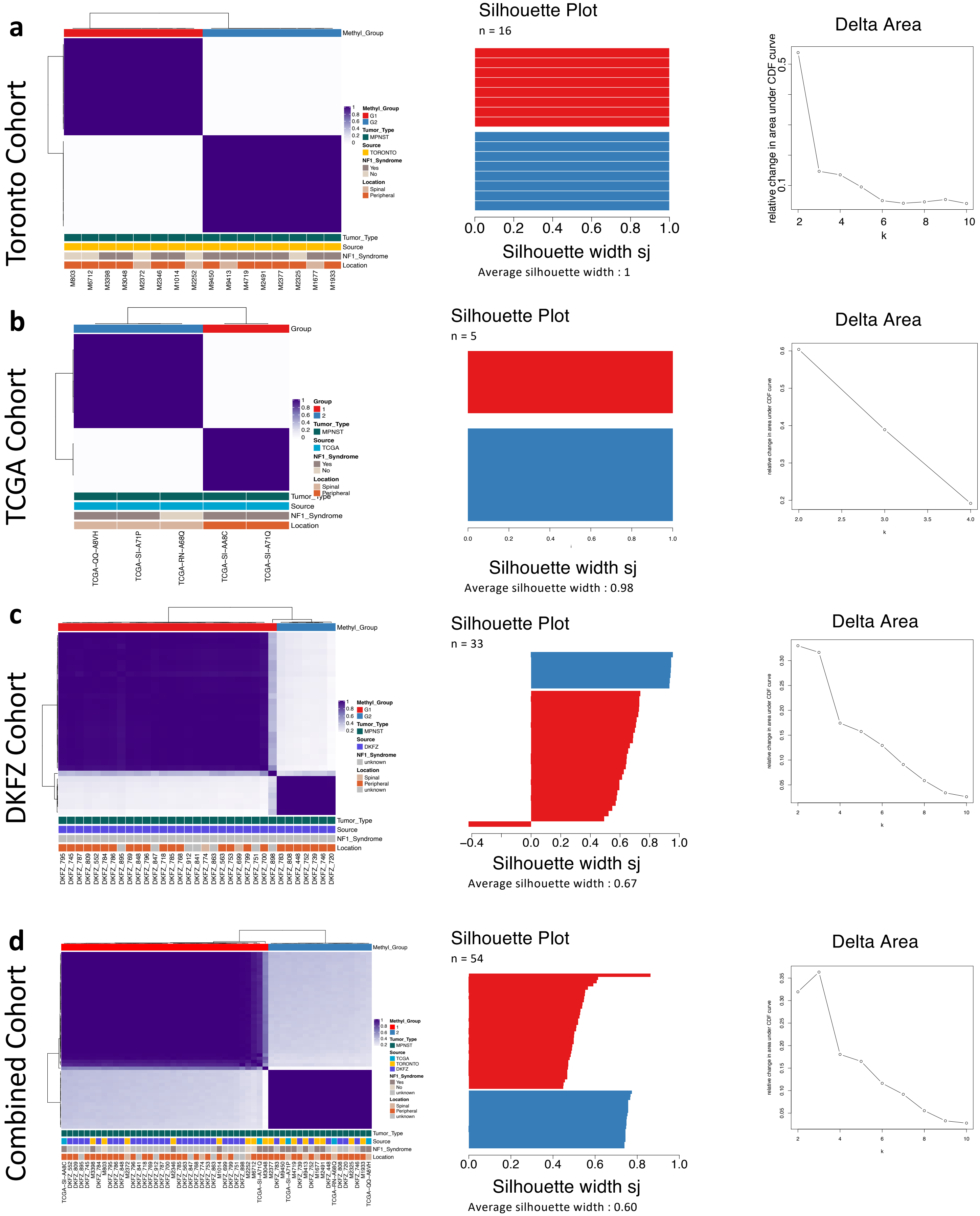
a
Toronto Cohort
Methyl_Group
Methyl_Group
G1
G2
Tumor_Type
MPNST
Source
TORONTO
NF1_Syndrome
Yes
No
Location
Spinal
Peripheral
Tumor_Type
Source
NF1_Syndrome
Location
M803 M6712 M3398 M3048 M2372 M2346 M1014 M2252 M9450 M9413 M4719 M2491 M2377 M2325 M1677 M1933
Silhouette Plot
n = 16
Silhouette width sj
Average silhouette width : 1
Delta Area
relative change in area under CDF curve
k
b
TCGA Cohort
Group
Tumor_Type
MPNST
Source
TCGA
NF1_Syndrome
Yes
No
Location
Spinal
Peripheral
TCGA–QQ–A8VH TCGA–SI–A71P TCGA–RN–A68Q TCGA–SI–AA8C TCGA–SI–A71Q
Silhouette Plot
n = 5
Silhouette width sj
Average silhouette width : 0.98
Delta Area
relative change in area under CDF curve
k
c
DKFZ Cohort
Methyl_Group
G1
G2
Tumor_Type
MPNST
Source
DKFZ
NF1_Syndrome
unknown
Location
Spinal
Peripheral
unknown
Silhouette Plot
n = 33
Silhouette width sj
Average silhouette width : 0.67
Delta Area
relative change in area under CDF curve
k
d
Combined Cohort
Methyl_Group
Tumor_Type
MPNST
Source
TCGA
TORONTO
DKFZ
NF1_Syndrome
Yes
No
unknown
Location
Spinal
Peripheral
unknown
Silhouette Plot
n = 54
Silhouette width sj
Average silhouette width : 0.60
Delta Area
relative change in area under CDF curve
k

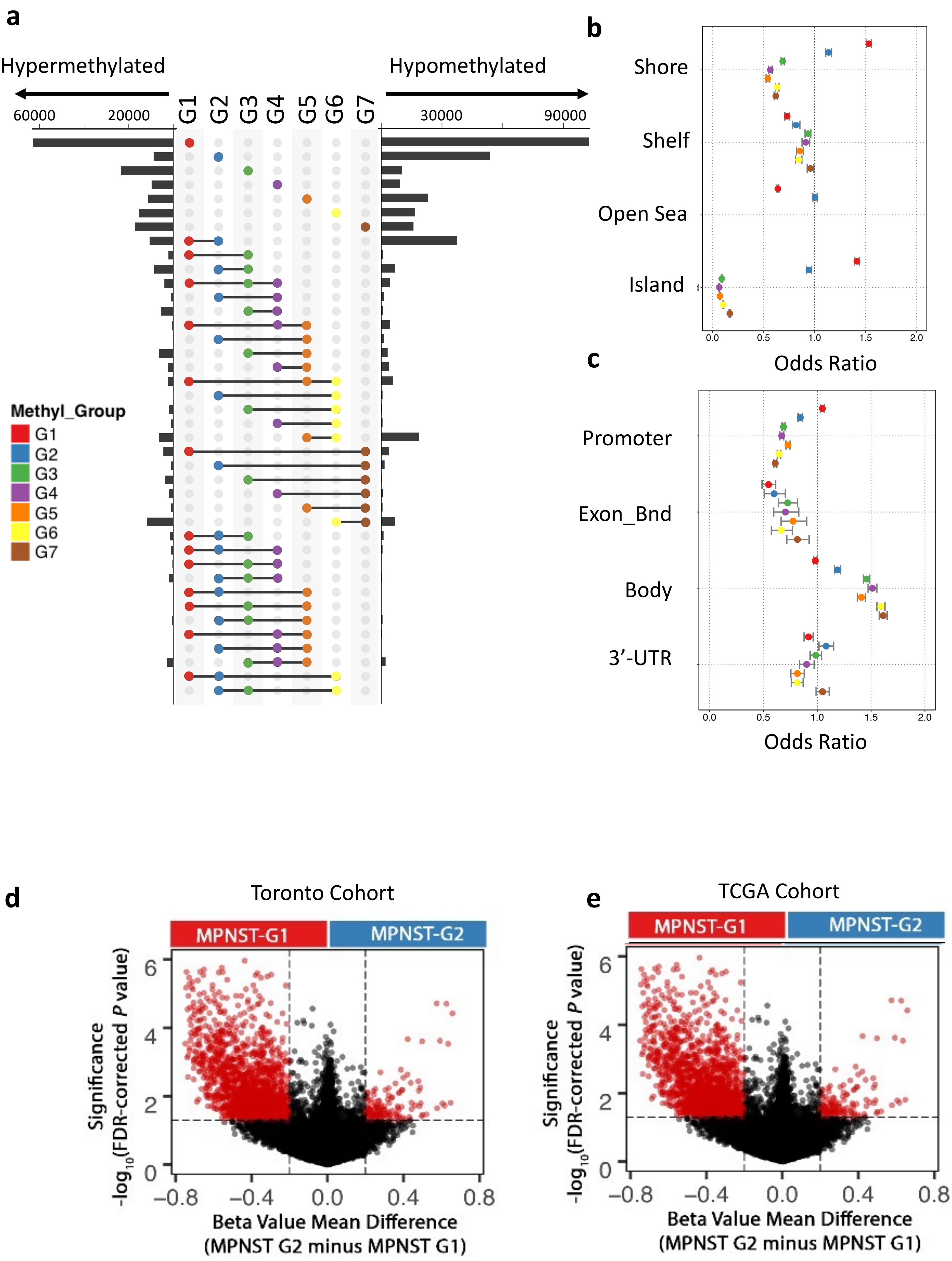
a
Hypermethylated
Hypomethylated
60000
20000
30000
90000
G1
G2
G3
G4
G5
G6
G7
Methyl_Group
G1
G2
G3
G4
G5
G6
G7
b
Shore
Shelf
Open Sea
Island
0.0
0.5
1.0
1.5
2.0
Odds Ratio
c
Promoter
Exon_Bnd
Body
3'-UTR
0.0
0.5
1.0
1.5
2.0
Odds Ratio
d
Toronto Cohort
MPNST-G1
MPNST-G2
Significance
-log10(FDR-corrected P value)
6
4
2
0
−0.8
−0.4
0.0
0.4
0.8
Beta Value Mean Difference
(MPNST G2 minus MPNST G1)
e
TCGA Cohort
MPNST-G1
MPNST-G2
Significance
-log10(FDR-corrected P value)
6
4
2
0
−0.8
−0.4
0.0
0.4
0.8
Beta Value Mean Difference
(MPNST G2 minus MPNST G1)

a
b
c
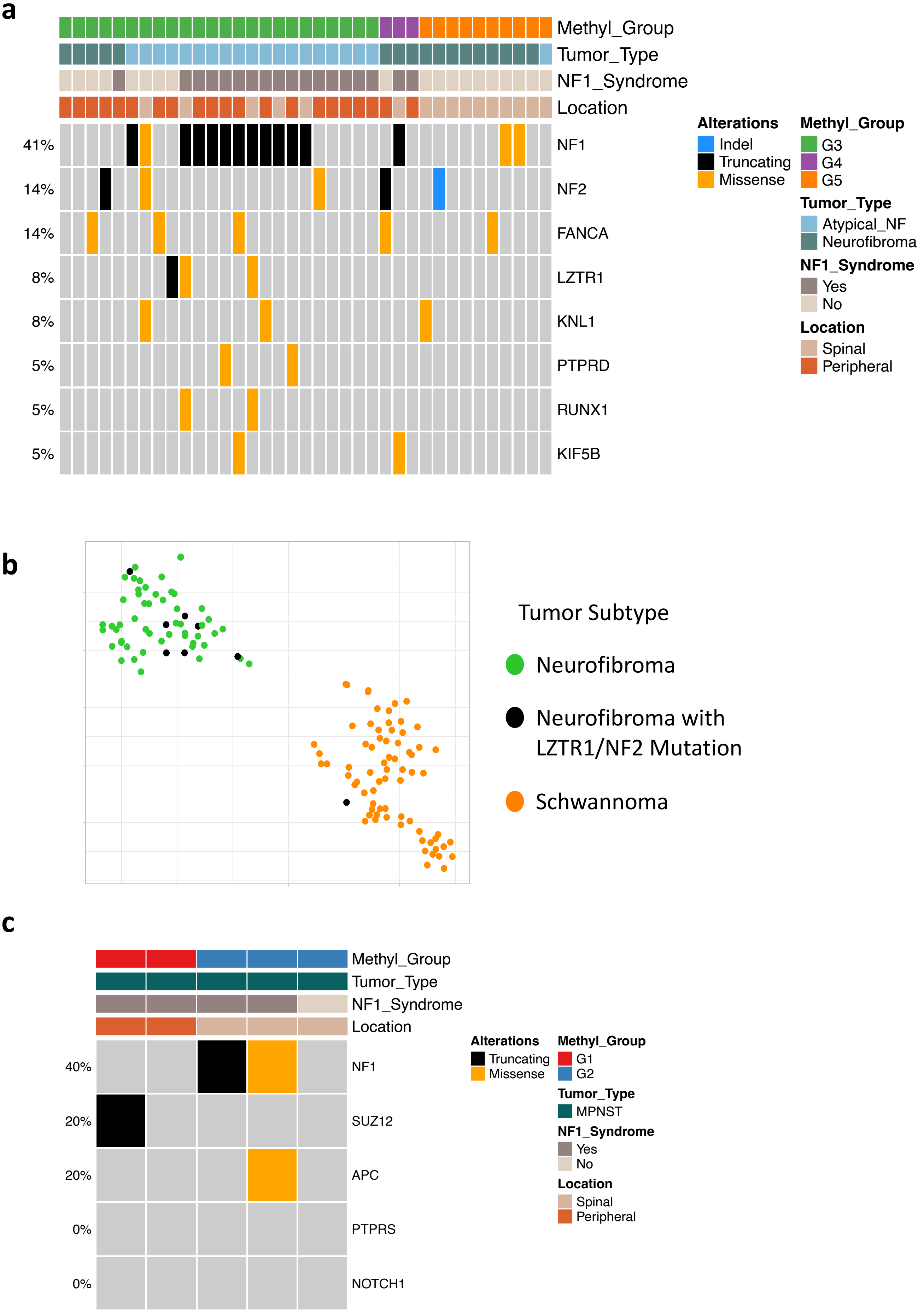
Methyl_Group
Tumor_Type
NF1_Syndrome
Location
41%
14%
14%
8%
8%
5%
5%
5%
NF1
NF2
FANCA
LZTR1
KNL1
PTPRD
RUNX1
KIF5B
Alterations
Indel
Truncating
Missense
Methyl_Group
G3
G4
G5
Tumor_Type
Atypical_NF
Neurofibroma
NF1_Syndrome
Yes
No
Location
Spinal
Peripheral
Tumor Subtype
Neurofibroma
Neurofibroma with LZTR1/NF2 Mutation
Schwannoma
Methyl_Group
Tumor_Type
NF1_Syndrome
Location
40%
20%
20%
0%
0%
NF1
SUZ12
APC
PTPRS
NOTCH1
Alterations
Truncating
Missense
Methyl_Group
G1
G2
Tumor_Type
MPNST
NF1_Syndrome
Yes
No
Location
Spinal
Peripheral

a

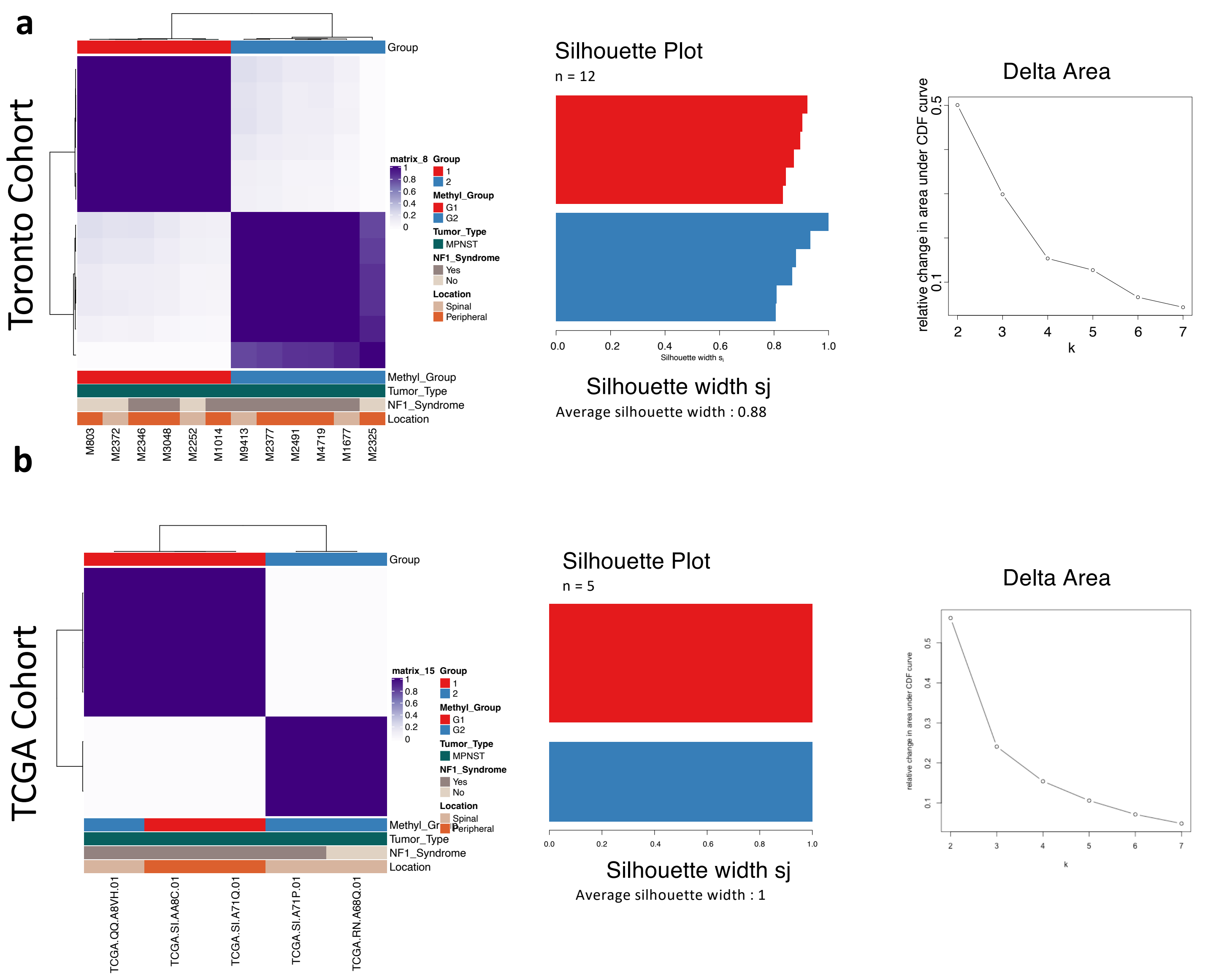

c

Clustering Based on Methylation Profile (columns) / **Clustering Based on Transcriptome Profile** (rows)

	MPNST-G1	MPNST-G2
MPNST-G1	8	1
MPNST-G2	0	8

Adjusted Rand Index = 0.76, $p < 0.001$, n=17

d

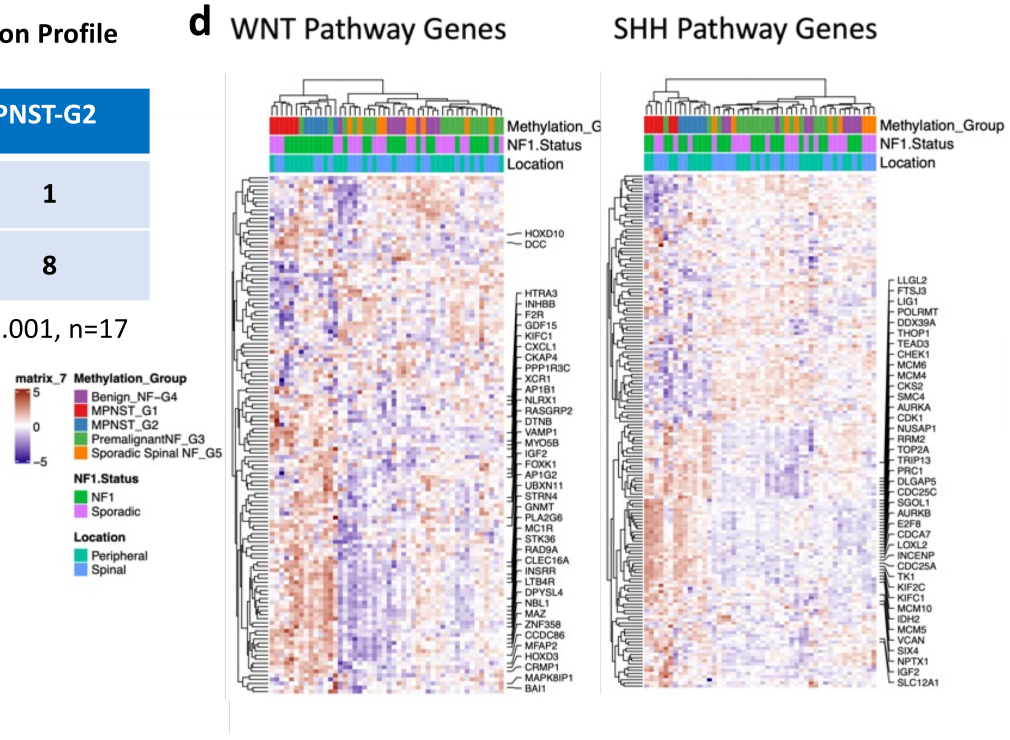

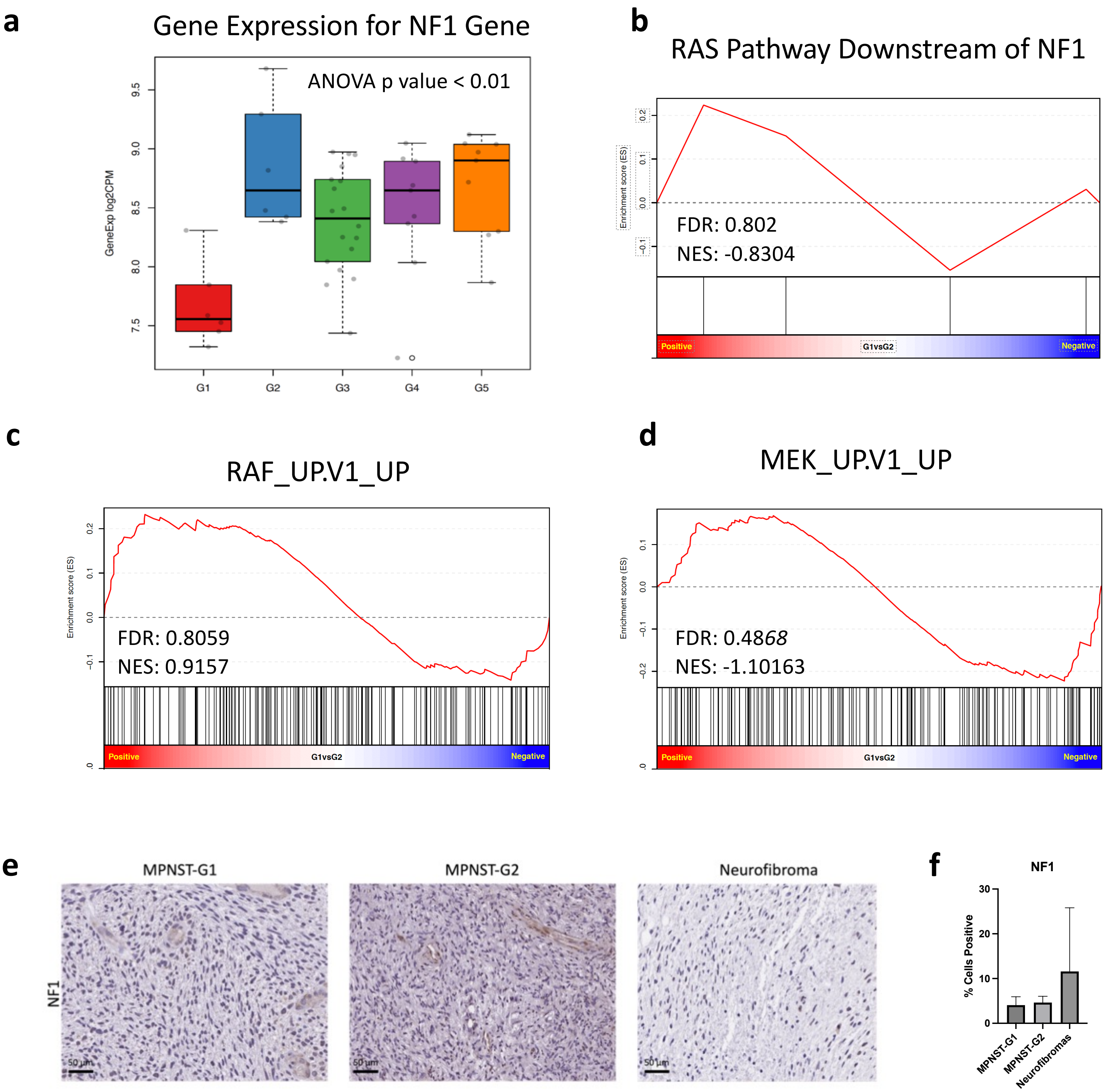
a
Gene Expression for NF1 Gene
ANOVA p value < 0.01
GeneExp log2CPM
7.5
8.0
8.5
9.0
9.5
G1
G2
G3
G4
G5
b
RAS Pathway Downstream of NF1
Enrichment score (ES)
0.2
0.1
0.0
-0.1
FDR: 0.802
NES: -0.8304
Positive
G1vsG2
Negative
c
RAF_UP.V1_UP
Enrichment score (ES)
0.2
0.1
0.0
-0.1
FDR: 0.8059
NES: 0.9157
Positive
G1vsG2
Negative
d
MEK_UP.V1_UP
Enrichment score (ES)
0.1
0.0
-0.1
-0.2
FDR: 0.4868
NES: -1.10163
Positive
G1vsG2
Negative
e
MPNST-G1
MPNST-G2
Neurofibroma
NF1
50 µm
f
NF1
% Cells Positive
0
10
20
30
MPNST-G1
MPNST-G2
Neurofibromas

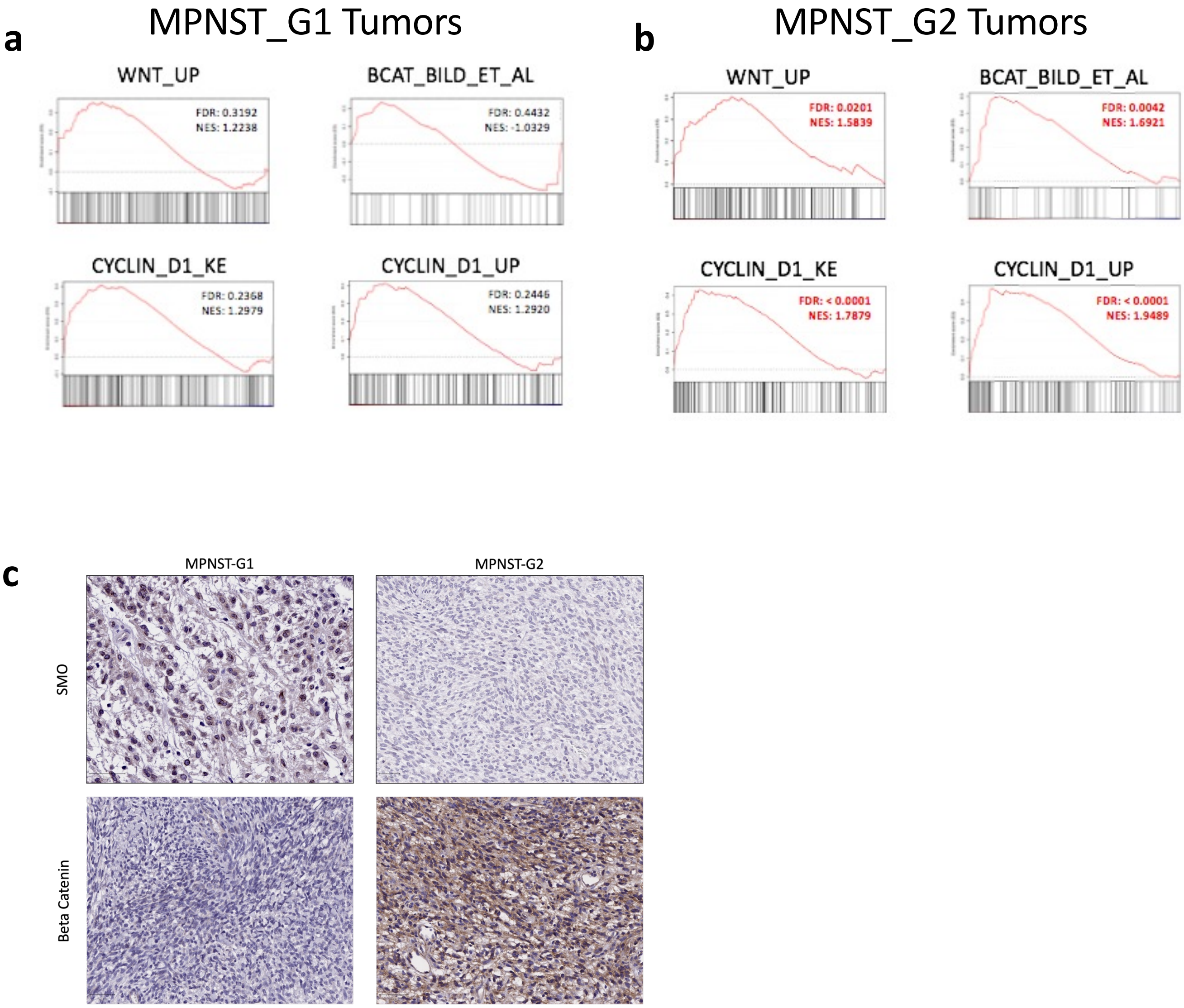
a
MPNST_G1 Tumors
WNT_UP
FDR: 0.3192
NES: 1.2238
BCAT_BILD_ET_AL
FDR: 0.4432
NES: -1.0329
CYCLIN_D1_KE
FDR: 0.2368
NES: 1.2979
CYCLIN_D1_UP
FDR: 0.2446
NES: 1.2920
b
MPNST_G2 Tumors
WNT_UP
FDR: 0.0201
NES: 1.5839
BCAT_BILD_ET_AL
FDR: 0.0042
NES: 1.6921
CYCLIN_D1_KE
FDR: < 0.0001
NES: 1.7879
CYCLIN_D1_UP
FDR: < 0.0001
NES: 1.9489
c
MPNST-G1
MPNST-G2
SMO
Beta Catenin

a

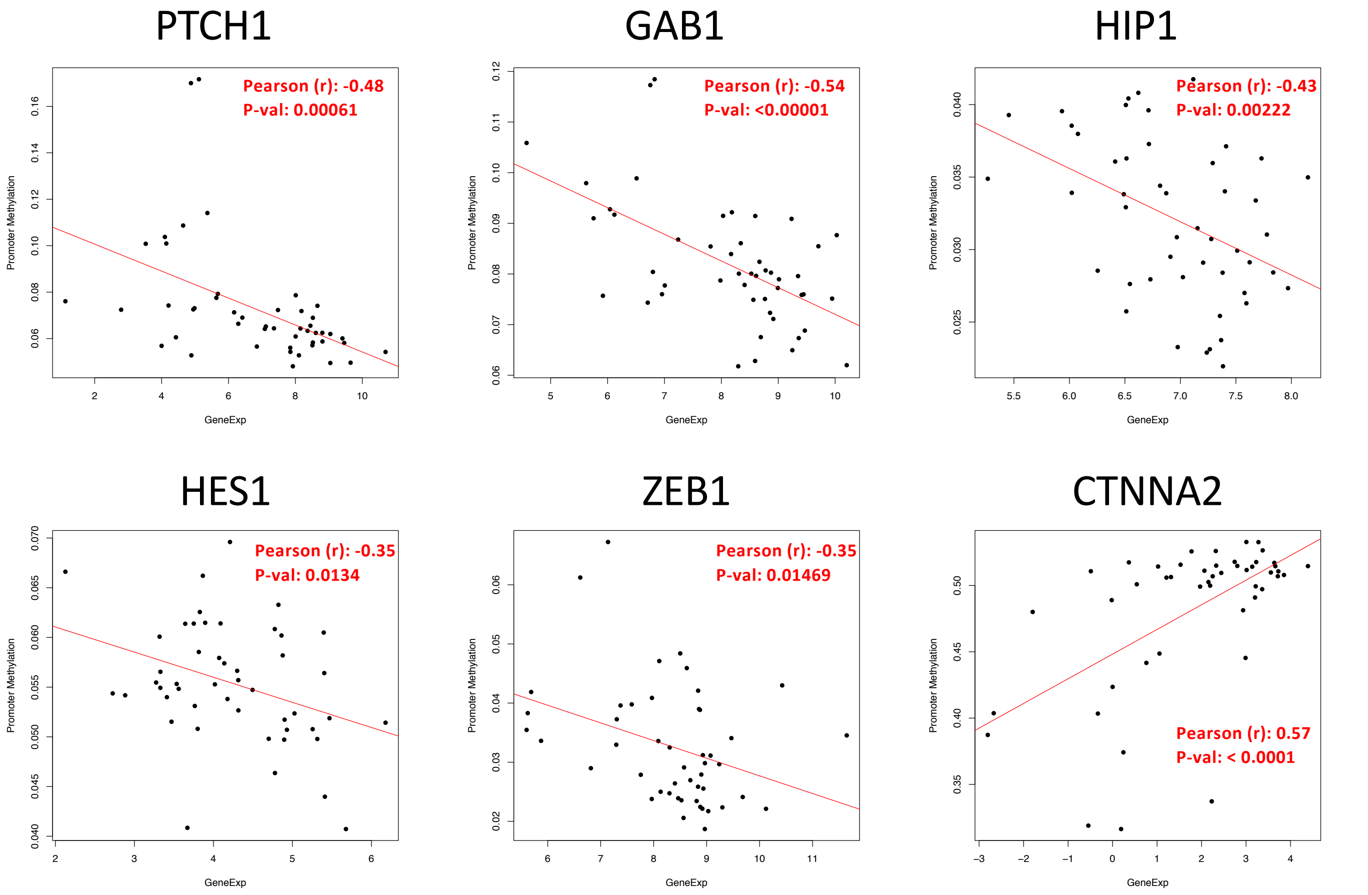
PTCH1
Pearson (r): -0.48
P-val: 0.00061
GAB1
Pearson (r): -0.54
P-val: <0.00001
HIP1
Pearson (r): -0.43
P-val: 0.00222
HES1
Pearson (r): -0.35
P-val: 0.0134
ZEB1
Pearson (r): -0.35
P-val: 0.01469
CTNNA2
Pearson (r): 0.57
P-val: < 0.0001
Promoter Methylation
GeneExp

b

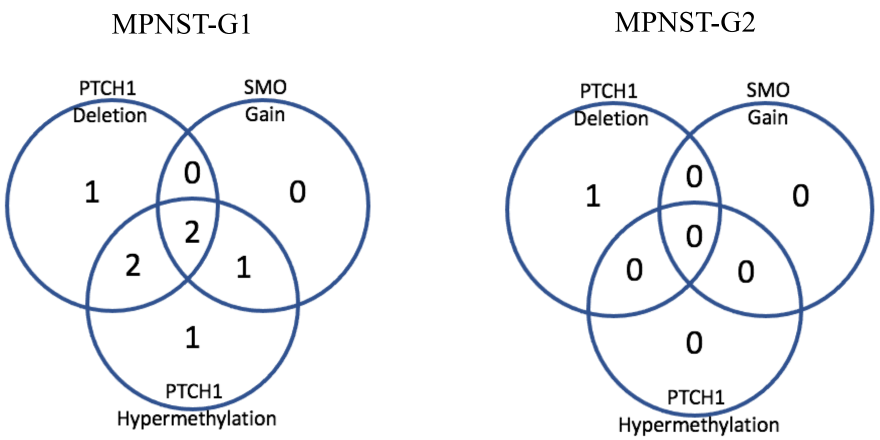
MPNST-G1
MPNST-G2
PTCH1 Deletion
SMO Gain
PTCH1 Hypermethylation
1
0
0
2
2
1
1
1
0
0
0
0
0
0

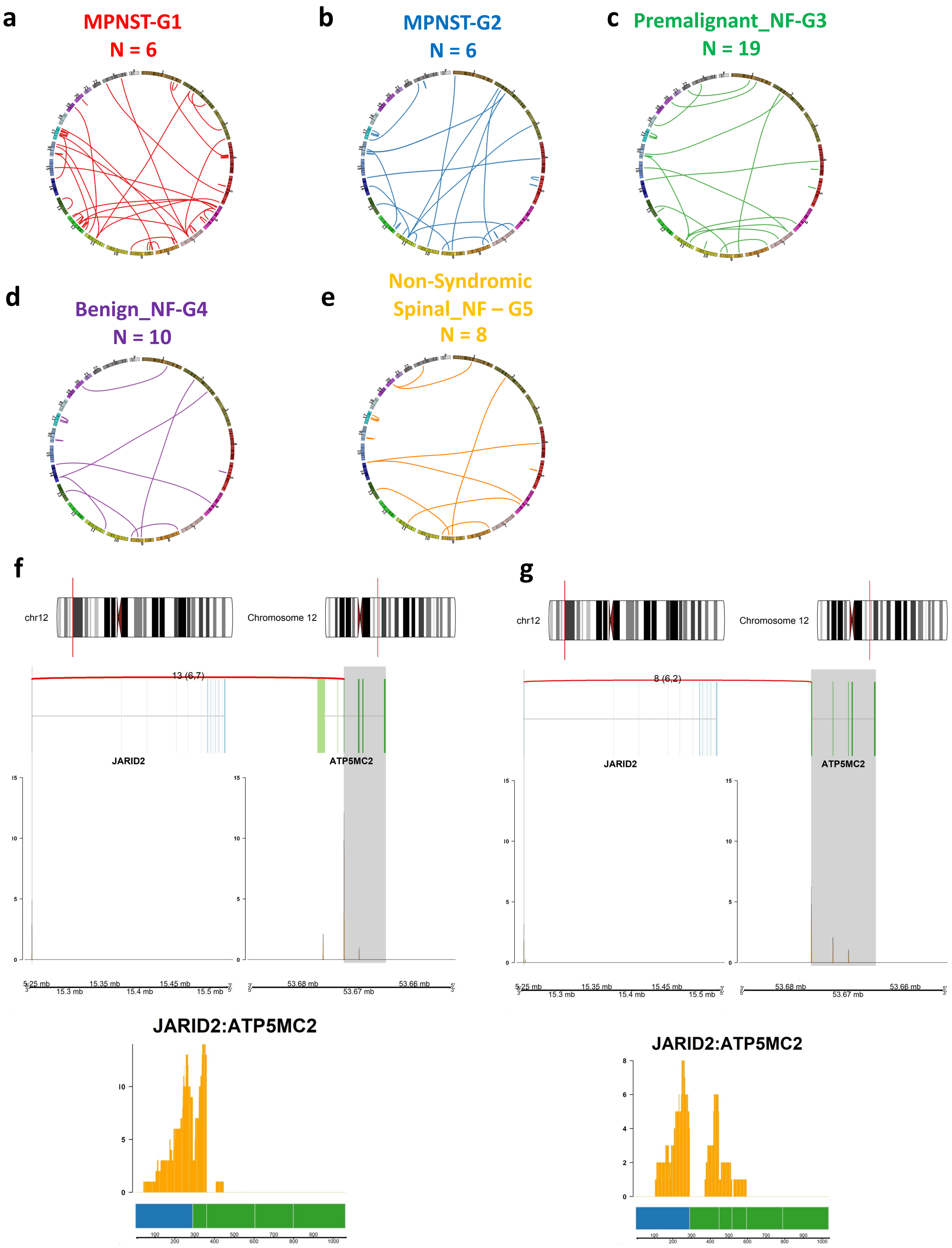
a
MPNST-G1
N = 6
b
MPNST-G2
N = 6
c
Premalignant_NF-G3
N = 19
d
Benign_NF-G4
N = 10
e
Non-Syndromic
Spinal_NF – G5
N = 8
f
chr12
Chromosome 12
13 (6,7)
JARID2
ATP5MC2
15.3 mb
15.35 mb
15.4 mb
15.45 mb
15.5 mb
53.68 mb
53.67 mb
53.66 mb
JARID2:ATP5MC2
g
chr12
Chromosome 12
8 (6,2)
JARID2
ATP5MC2
JARID2:ATP5MC2

a

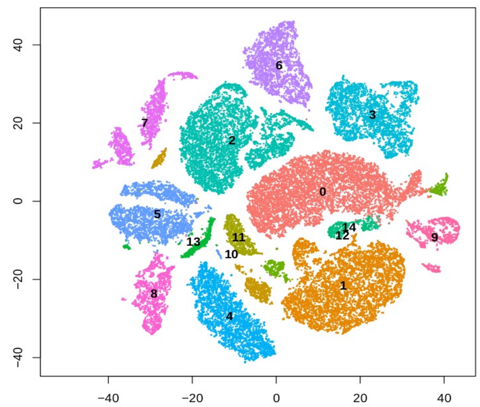
40
20
0
−20
−40
−40 −20 0 20 40
0 1 2 3 4 5 6 7 8 9 10 11 12 13 14

b

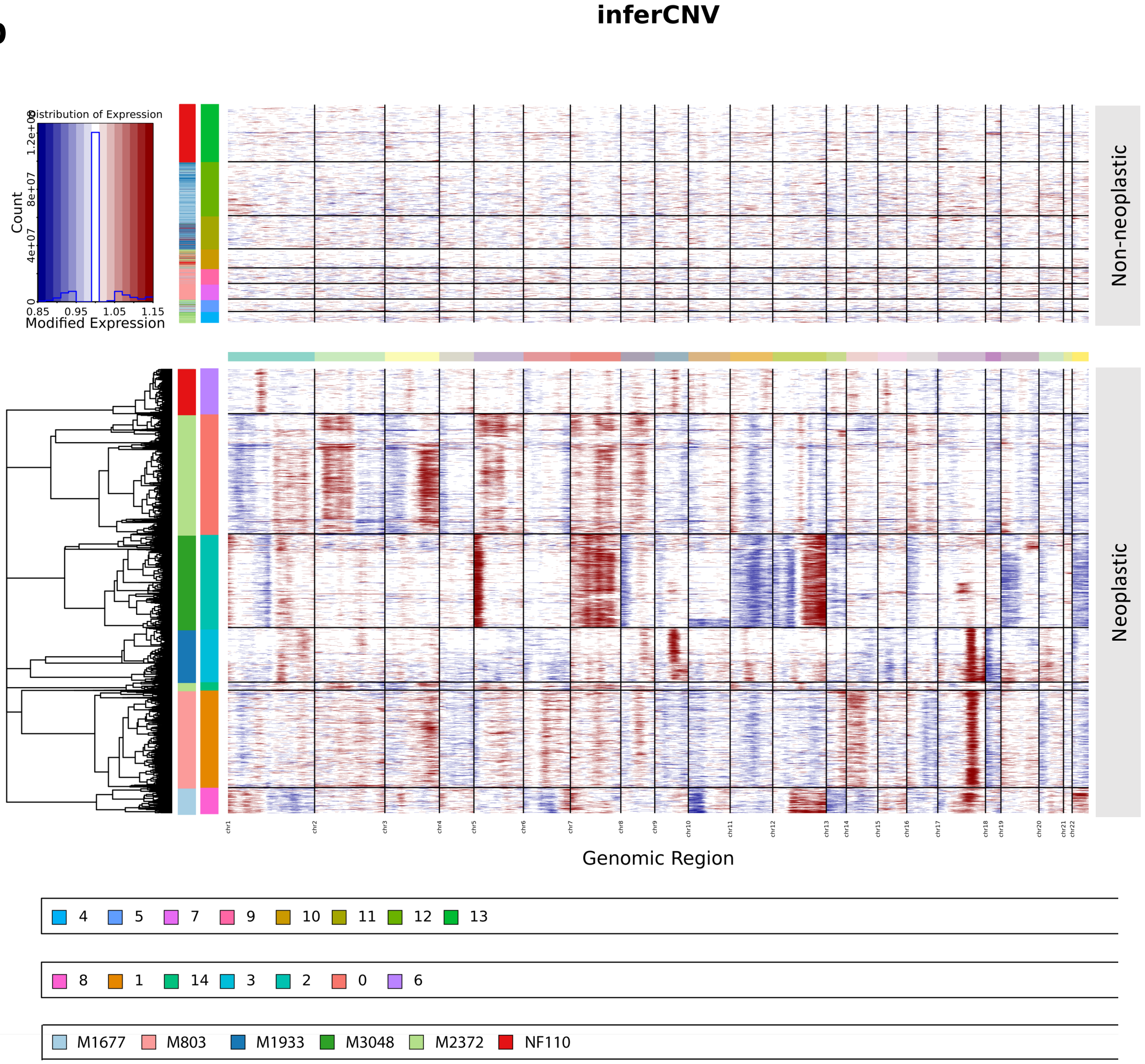
inferCNV
Distribution of Expression
Count
Modified Expression
0.85 0.95 1.05 1.15
Non-neoplastic
Neoplastic
chr1 chr2 chr3 chr4 chr5 chr6 chr7 chr8 chr9 chr10 chr11 chr12 chr13 chr14 chr15 chr16 chr17 chr18 chr19 chr20 chr21 chr22
Genomic Region
4 5 7 9 10 11 12 13
8 1 14 3 2 0 6
M1677 M803 M1933 M3048 M2372 NF110

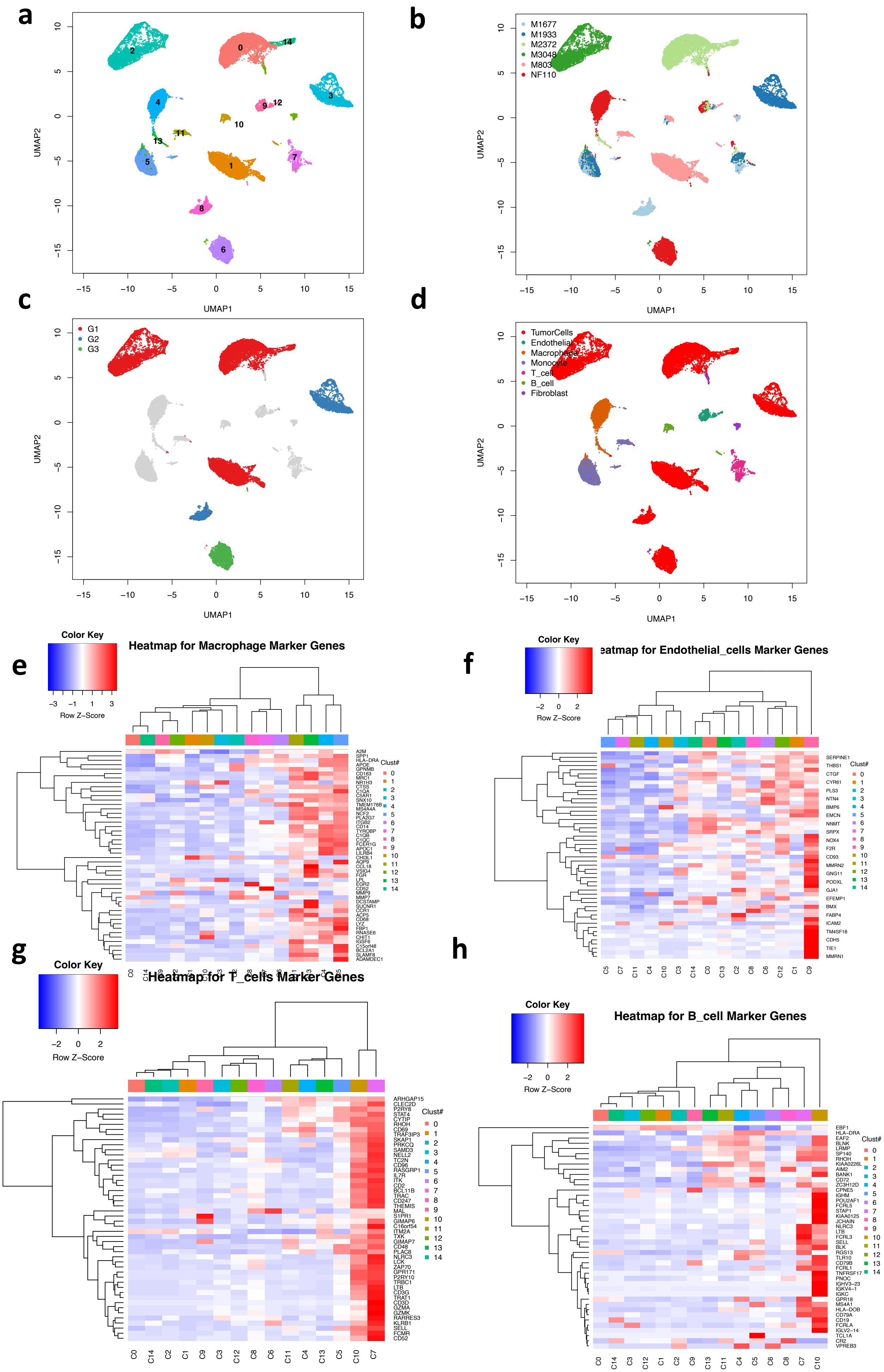
a
b
c
d
e
f
g
h
UMAP1
UMAP2
M1677
M1933
M2372
M3048
M803
NF110
G1
G2
G3
TumorCells
Endothelial
Macrophage
Monocyte
T_cell
B_cell
Fibroblast
Color Key
Row Z−Score
Heatmap for Macrophage Marker Genes
eatmap for Endothelial_cells Marker Genes
Heatmap for T_cells Marker Genes
Heatmap for B_cell Marker Genes
Clust#

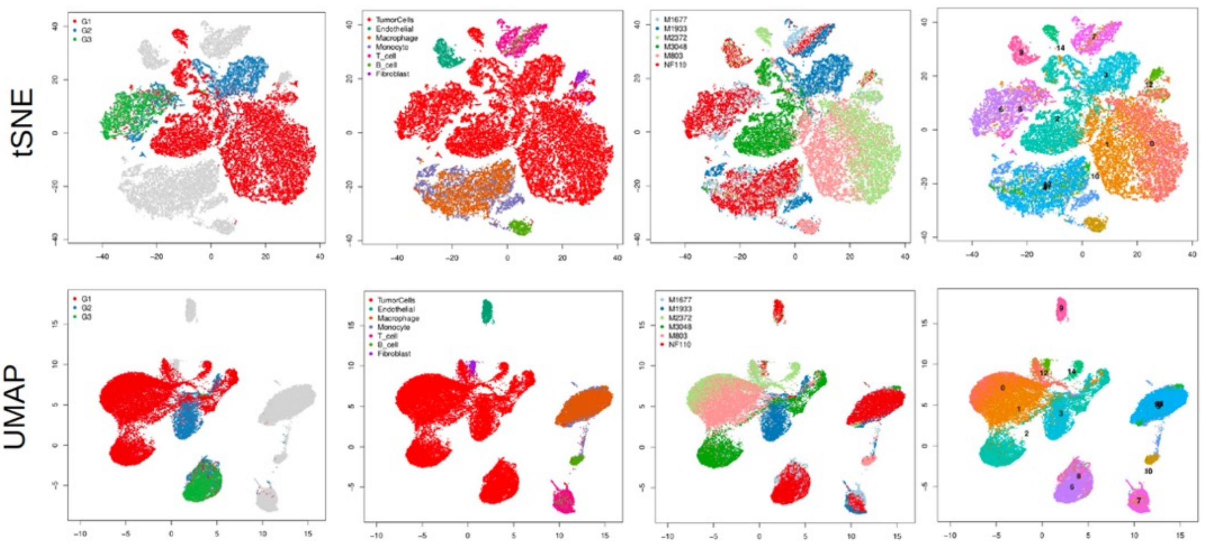
tSNE
UMAP
G1
G2
G3
TumorCells
Endothelial
Macrophage
Monocyte
T_cell
B_cell
Fibroblast
M1677
M1933
M2372
M3048
M803
NF110

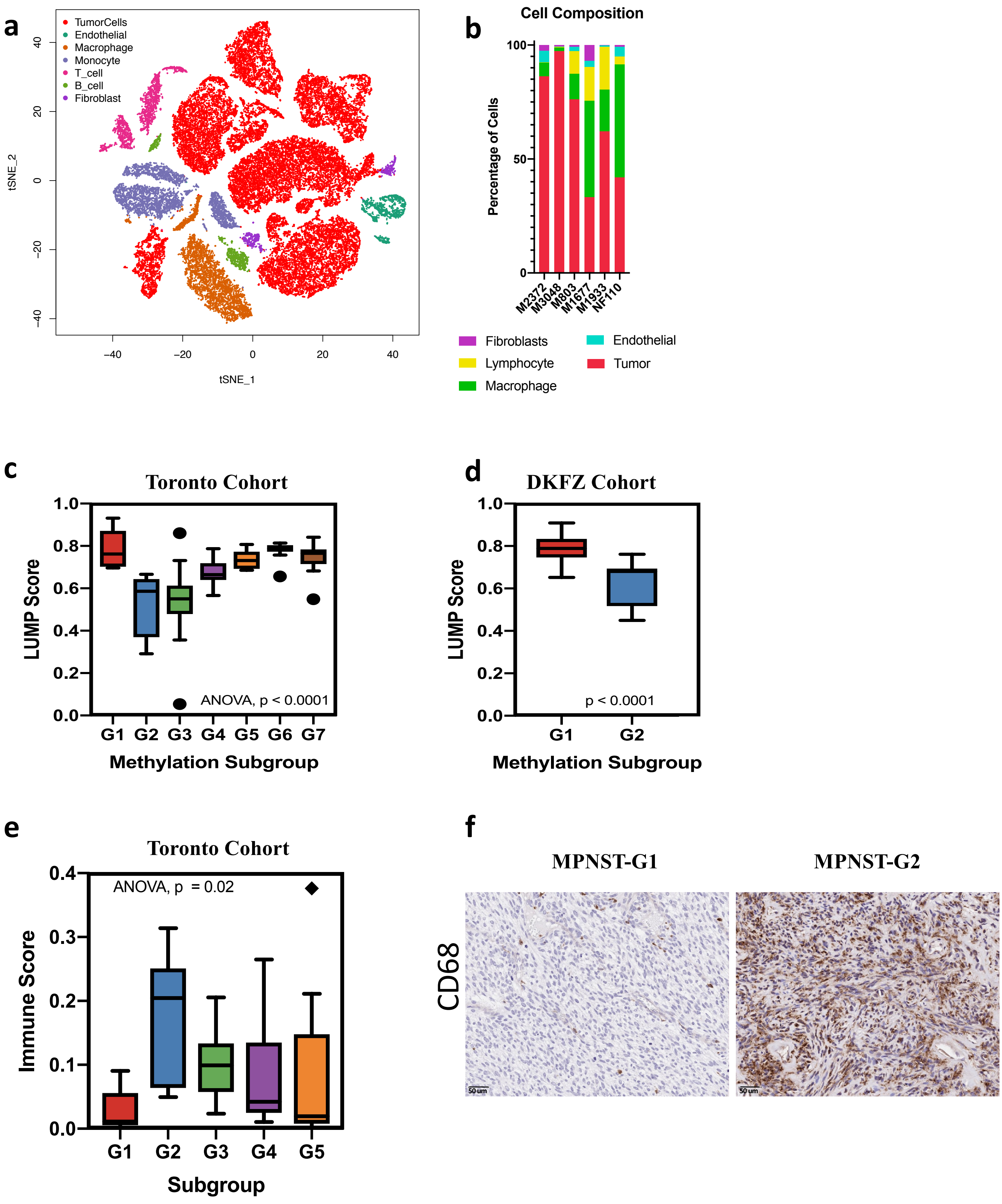
a
TumorCells
Endothelial
Macrophage
Monocyte
T_cell
B_cell
Fibroblast
tSNE_2
tSNE_1
b
Cell Composition
Percentage of Cells
M2372
M3048
M803
M1677
M1933
NF110
Fibroblasts
Endothelial
Lymphocyte
Tumor
Macrophage
c
Toronto Cohort
LUMP Score
ANOVA, p < 0.0001
G1 G2 G3 G4 G5 G6 G7
Methylation Subgroup
d
DKFZ Cohort
LUMP Score
p < 0.0001
G1 G2
Methylation Subgroup
e
Toronto Cohort
Immune Score
ANOVA, p = 0.02
G1 G2 G3 G4 G5
Subgroup
f
MPNST-G1
MPNST-G2
CD68
50 um
50 um

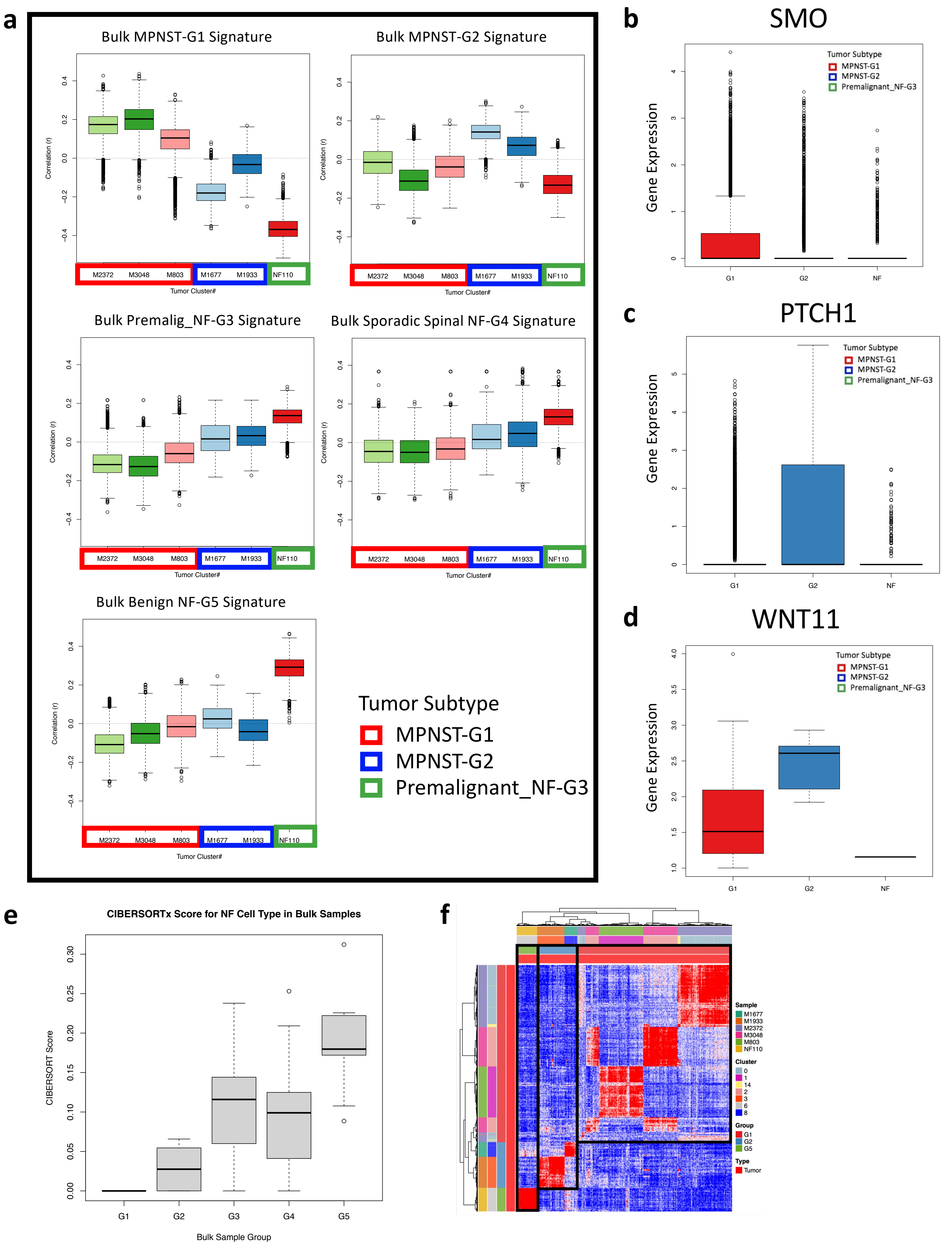
a
Bulk MPNST-G1 Signature
Bulk MPNST-G2 Signature
Bulk Premalig_NF-G3 Signature
Bulk Sporadic Spinal NF-G4 Signature
Bulk Benign NF-G5 Signature
Correlation (r)
M2372
M3048
M803
M1677
M1933
NF110
Tumor Cluster#
Tumor Subtype
MPNST-G1
MPNST-G2
Premalignant_NF-G3
b
SMO
c
PTCH1
d
WNT11
Gene Expression
G1
G2
NF
e
CIBERSORTx Score for NF Cell Type in Bulk Samples
CIBERSORT Score
Bulk Sample Group
G1
G2
G3
G4
G5
f
Sample
M1677
M1933
M2372
M3048
M803
NF110
Cluster
Group
G1
G2
G5
Type
Tumor

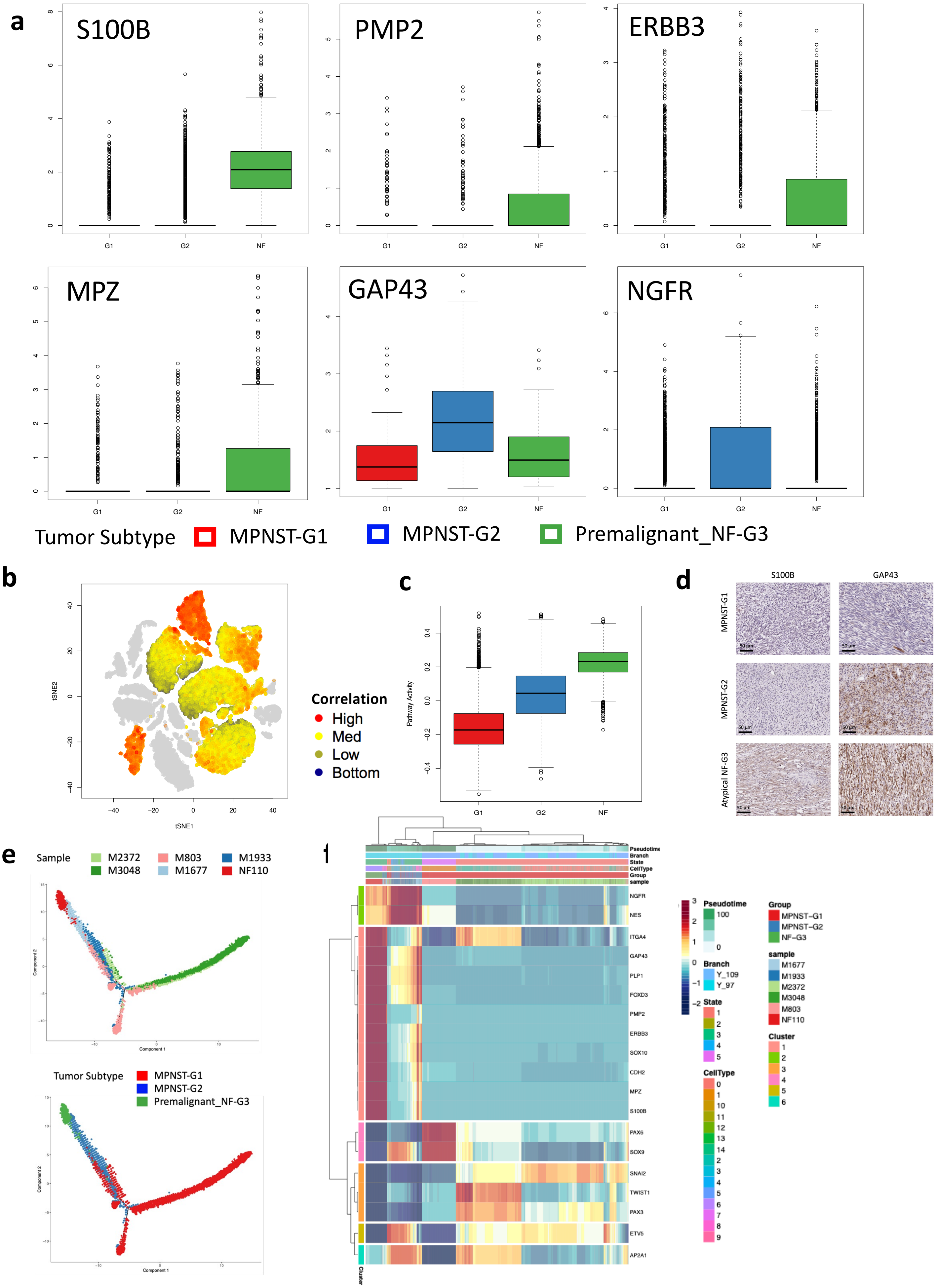
a
S100B
PMP2
ERBB3
MPZ
GAP43
NGFR
G1
G2
NF
Tumor Subtype
MPNST-G1
MPNST-G2
Premalignant_NF-G3
b
tSNE1
tSNE2
Correlation
High
Med
Low
Bottom
c
Pathway Activity
d
S100B
GAP43
MPNST-G1
MPNST-G2
Atypical NF-G3
e
Sample
M2372
M3048
M803
M1677
M1933
NF110
Component 1
Component 2
Tumor Subtype
MPNST-G1
MPNST-G2
Premalignant_NF-G3
f
Pseudotime
Branch
State
CellType
Group
sample
NGFR
NES
ITGA4
GAP43
PLP1
FOXD3
PMP2
ERBB3
SOX10
CDH2
MPZ
S100B
PAX6
SOX9
SNAI2
TWIST1
PAX3
ETV5
AP2A1
Cluster
Pseudotime
100
0
Branch
Y_109
Y_97
State
CellType
Group
MPNST−G1
MPNST−G2
NF−G3
sample
M1677
M1933
M2372
M3048
M803
NF110
Cluster

a

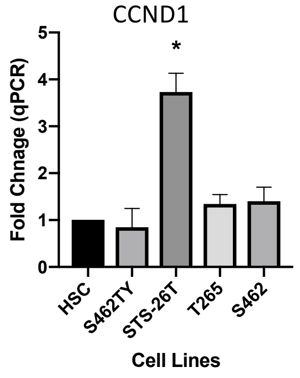

CCND1
*
Fold Chnage (qPCR)
0
1
2
3
4
5
HSC
S462TY
STS-26T
T265
S462
Cell Lines

b

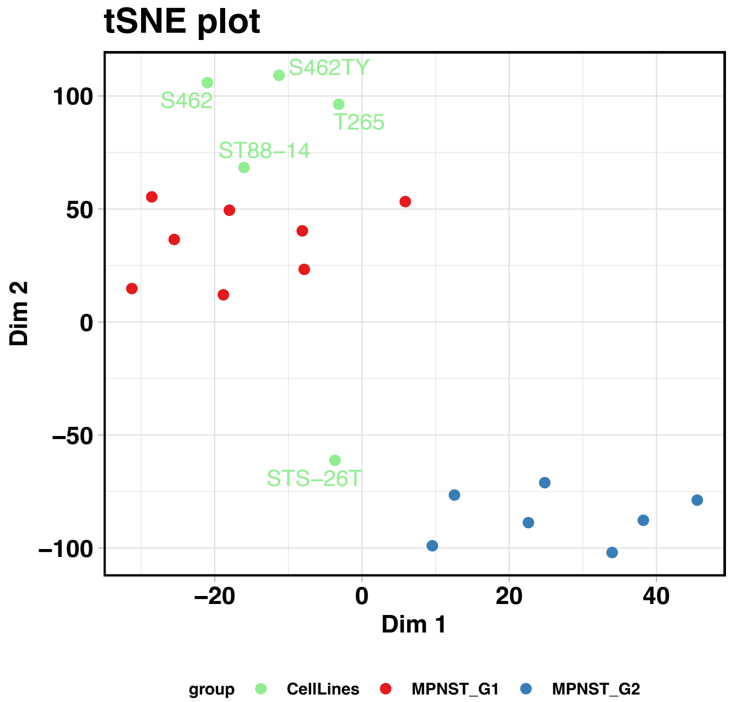

tSNE plot
S462TY
S462
T265
ST88−14
STS−26T
100
50
0
−50
−100
Dim 2
−20
0
20
40
Dim 1
group
CellLines
MPNST_G1
MPNST_G2

a

Xenograft Tumor Formation in NRG Mice

Cell Line	Mice Injected	Tumors Formed
HSC1λ g*GFP*	4	0
HSC1λ g*PTCH1*	4	3
HSC1λ *NF1-/-*; g*PTCH1*	4	4
HSC1λ *NF1-/-*	12	6

b

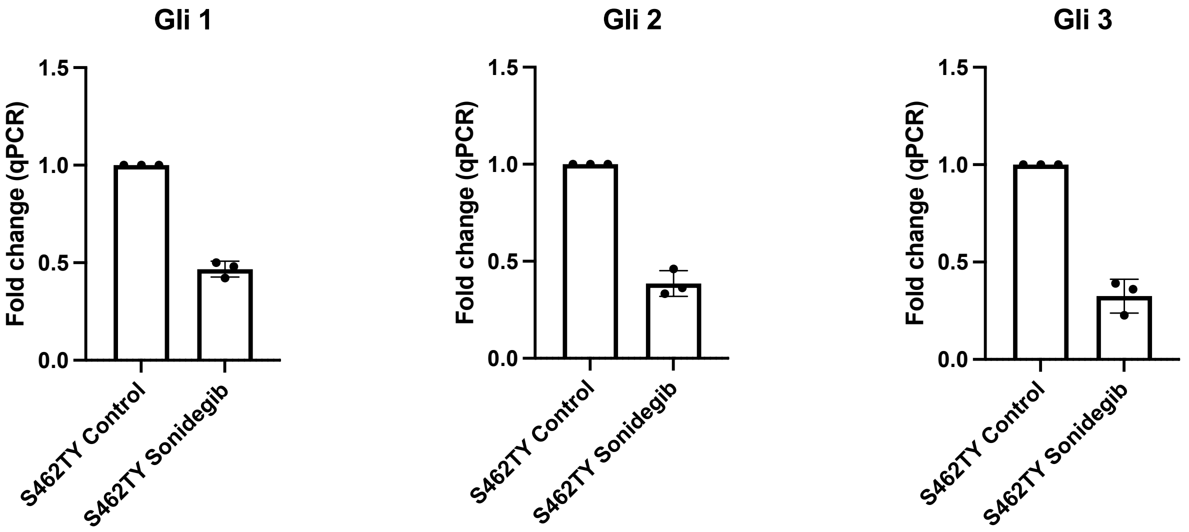

c

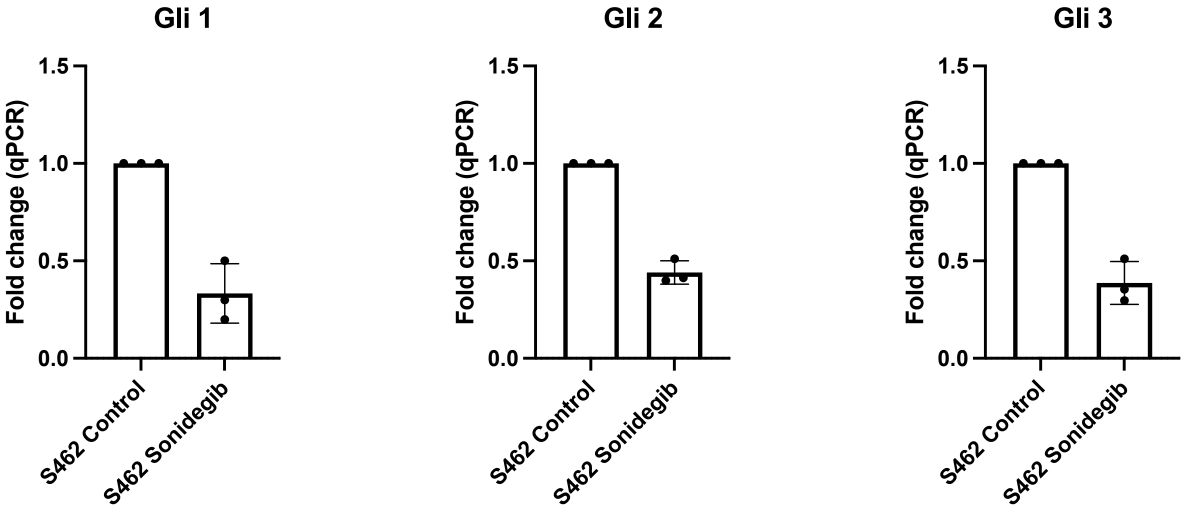

Supplement: Supplementary file 1 — Supplementary Information [file 41467_2023_38432_MOESM1_ESM.pdf]
