# Supplementary material for: Multiplatform molecular profiling uncovers two subgroups of malignant peripheral nerve sheath tumors with distinct therapeutic vulnerabilities
Source: Nat Commun. 2023 May 10;14:2696. doi: 10.1038/s41467-023-38432-6 (PMC10172395; doi:10.1038/s41467-023-38432-6)
Supplement: Supplementary file 2 — Reporting Summary [file 41467_2023_38432_MOESM2_ESM.pdf]

nature portfolio

Corresponding author(s): Gelareh Zadeh

Last updated by author(s): April 1, 2023

Reporting Summary

Nature Portfolio wishes to improve the reproducibility of the work that we publish. This form provides structure for consistency and transparency in reporting. For further information on Nature Portfolio policies, see our Editorial Policies and the Editorial Policy Checklist.

Statistics

For all statistical analyses, confirm that the following items are present in the figure legend, table legend, main text, or Methods section.

n/a	Confirmed	
☐	☒	The exact sample size (n) for each experimental group/condition, given as a discrete number and unit of measurement
☐	☒	A statement on whether measurements were taken from distinct samples or whether the same sample was measured repeatedly
☐	☒	The statistical test(s) used AND whether they are one- or two-sided<br>*Only common tests should be described solely by name; describe more complex techniques in the Methods section.*
☐	☒	A description of all covariates tested
☐	☒	A description of any assumptions or corrections, such as tests of normality and adjustment for multiple comparisons
☐	☒	A full description of the statistical parameters including central tendency (e.g. means) or other basic estimates (e.g. regression coefficient) AND variation (e.g. standard deviation) or associated estimates of uncertainty (e.g. confidence intervals)
☐	☒	For null hypothesis testing, the test statistic (e.g. F, t, r) with confidence intervals, effect sizes, degrees of freedom and P value noted<br>*Give P values as exact values whenever suitable.*
☒	☐	For Bayesian analysis, information on the choice of priors and Markov chain Monte Carlo settings
☐	☒	For hierarchical and complex designs, identification of the appropriate level for tests and full reporting of outcomes
☒	☐	Estimates of effect sizes (e.g. Cohen's d, Pearson's r), indicating how they were calculated

Our web collection on statistics for biologists contains articles on many of the points above.

Software and code

Policy information about availability of computer code

Data collection	No software was used

Data analysis	Picard v1.72<br>GATK v3.6.0<br>Mutect V1.1.6<br>Strelka v1.0.13<br>Mutect2 V1.1<br>Variant Effect Predictor v.92.0<br>STAR aligner (v.2.6.0)<br>Rsubread (v.1.5.0)<br>edgeR(v.3.22.3)<br>FusionCatcher v1.1.0<br>Bowtie (v1.2)<br>Bowtie2 (v2.3)<br>BLAT (v0.35)<br>Suerat version 3.0<br>inferCNV (v.1.1.1)<br>MONOCLE2<br>DDRTree (V.01)<br>Harmony (v.01)

For manuscripts utilizing custom algorithms or software that are central to the research but not yet described in published literature, software must be made available to editors and reviewers. We strongly encourage code deposition in a community repository (e.g. GitHub). See the Nature Portfolio guidelines for submitting code & software for further information.

Data

Policy information about availability of data

All manuscripts must include a data availability statement. This statement should provide the following information, where applicable:

- Accession codes, unique identifiers, or web links for publicly available datasets
- A description of any restrictions on data availability
- For clinical datasets or third party data, please ensure that the statement adheres to our policy

Raw sequencing data for all datatypes have been deposited into public repositories. Methylation (idat), bulk mRNA (fastq) and snRNA (fastq) datasets has been deposited to the Gene Expression Omnibus (GEO; GSE207207). Whole exome sequencing (fastq) has been deposited to Sequence Read Archive (PRJNA854920). Source data are provided as Source Data file.

Field-specific reporting

Please select the one below that is the best fit for your research. If you are not sure, read the appropriate sections before making your selection.

☒ Life sciences ☐ Behavioural & social sciences ☐ Ecological, evolutionary & environmental sciences

For a reference copy of the document with all sections, see nature.com/documents/nr-reporting-summary-flat.pdf

Life sciences study design

All studies must disclose on these points even when the disclosure is negative.

Sample size	Sample selection was performed retrospectively and determined based on availability of tissue and clinical data. Sample sizes were sufficient for clustering analyses used in this report. For all in vitro and in vivo studies a minimum of 3 biologically independent experiments were performed.
Data exclusions	One sample for RNA sequencing was excluded due to poor data quality.
Replication	Sequencing and molecular profiling was performed once per human sample because of the limited amount of tissue that is available per patient. All experiments were reliably reproduced and indicated in figure legends.
Randomization	No randomization was performed as tumors were collected retrospectively.
Blinding	Samples were blinded (histopathological diagnosis and patient outcomes) during the sample preparation and sequencing. The investigators performing analysis of IHC, western blots, mouse tumor measurements were blinded to condition.

Reporting for specific materials, systems and methods

We require information from authors about some types of materials, experimental systems and methods used in many studies. Here, indicate whether each material, system or method listed is relevant to your study. If you are not sure if a list item applies to your research, read the appropriate section before selecting a response.

Materials & experimental systems

n/a	Involved in the study
	[x] Antibodies
	[x] Eukaryotic cell lines
[x]	[ ] Palaeontology and archaeology
	[x] Animals and other organisms
	[x] Human research participants
[x]	[ ] Clinical data
[x]	[ ] Dual use research of concern

Methods

n/a	Involved in the study
[x]	[ ] ChIP-seq
[x]	[ ] Flow cytometry
[x]	[ ] MRI-based neuroimaging

Antibodies

Antibodies used	B-Actin(1:1000, Cat #8457S, Cell Signalling Technologies), Vinculin(1:30000, Cat #V9264, Sigma Aldrich), PTCH1 (1:500,Cat#MAB41051, R&D systems) and APC(1:500, Cat #15270, Abcam)<br>B-Actin (Cell Signaling Technologies #8457S, 1:10), PTCH1 (Cell Signaling Technologies #2468, 1:10), GLI1 (Cell Signaling Technologies #3538, 1:10), GAP43(1:500, #AB75810, Abcam), S100B (1:500, AB52642, Abcam), beta catenin (1:500, Cell Signaling Technologies #8408S), SMO (1:500, sc-13943, Santa Cruz Biotechnology).
Validation	B-Actin(1:1000, Cat #8457S, Cell Signalling Technologies) - https://www.cellsignal.com/products/primary-antibodies/b-actin-d6a8-rabbit-mab/8457<br>Vinculin(1:30000, Cat #V9264, Sigma Aldrich) - https://www.sigmaaldrich.com/CA/en/search/v9264?focus=products&page=1&perPage=30&sort=relevance&term=v9264&type=product_name<br>PTCH1(1:500,Cat#MAB41051, R&D systems) - https://www.rndsystems.com/products/human-mouse-patched-1-ptch-first-extracellular-loop-antibody-413220_mab41051<br>APC(1:500, Cat #15270, Abcam) - https://www.abcam.com/apc-antibody-ab15270.html<br>B-Actin (Cell Signaling Technologies #8457S, 1:10) - https://www.cellsignal.com/products/primary-antibodies/b-actin-d6a8-rabbit-mab/8457<br>PTCH1 (Cell Signaling Technologies #2468, 1:10) - https://www.cellsignal.com/products/primary-antibodies/ptch1-c53a3-rabbit-mab/2468<br>GLI1 (Cell Signaling Technologies #3538, 1:10) - https://www.cellsignal.com/products/primary-antibodies/gli1-c68h3-rabbit-mab/3538<br>SMO - https://www.nature.com/articles/onc201791#Sec9<br>Beta Catenin - https://www.cellsignal.com/products/primary-antibodies/b-catenin-d10a8-xp-rabbit-mab/8480<br>S100B - https://www.abcam.com/s100-beta-antibody-ep1576y-astrocyte-marker-ab52642.html<br>GAP43 -https://www.abcam.com/gap43-antibody-ep890y-ab75810.html

Eukaryotic cell lines

Policy information about cell lines

Cell line source(s)	Immortalized human Schwann cell (HSC1) and immortalized neurofibroma cell (ipNF06.2A28) were obtained from Dr. Margaret Wallace's laboratory at the University of Florida. MPNST cell lines (STS-26T, S462, S462TY , T265, 293T) were obtained from Dr. David Largaespada's laboratory at the University of Minnesota.
Authentication	Methylation profiling was performed on all cell lines.
Mycoplasma contamination	All cell lines were tested and were negative for mycoplasma contamination
Commonly misidentified lines (See ICLAC register)	No commonly misidentified cell lines were used.

Animals and other organisms

Policy information about studies involving animals; ARRIVE guidelines recommended for reporting animal research

Laboratory animals	Immunodeficient NOD-Rag1null IL2rgnull male mice (The Jackson Laboratory). Mice were 6-8 weeks of age.
Wild animals	Study did not involve wild animals.
Field-collected samples	Study did not involve filed-collected samples,
Ethics oversight	The University Health Network Animal Care committees approved this study protocol (AUP5935 and 6746). Animal care followed the guidelines of UHN Research Institutes' policies and the guidelines of the Canadian Council on Animal Care.

Note that full information on the approval of the study protocol must also be provided in the manuscript.

Human research participants

Policy information about studies involving human research participants

Population characteristics	Baseline population characteristics of patients with nerve sheath tumors (age, sex, history of neurofibromatosis, WHO grade, extent of surgical resection, adjuvant radiotherapy, tumor recurrence, median follow-up, anatomical location) are detailed in Supplementary Tables 1.
Recruitment	In this retrospective cohort, eligible cases were patients with pathologically confirmed nerve sheath tumor, confirmed by institutional neuropathologist with samples available from institutional biobanks and all clinical data above available. Participants were consented for institutional biobanking of blood and tissue samples for research at the time of surgery
Ethics oversight	University Health Network Research Ethics Board

Note that full information on the approval of the study protocol must also be provided in the manuscript.
