# Supplementary material for: Multiplatform molecular profiling uncovers two subgroups of malignant peripheral nerve sheath tumors with distinct therapeutic vulnerabilities
Source: Nat Commun. 2023 May 10;14:2696. doi: 10.1038/s41467-023-38432-6 (PMC10172395; doi:10.1038/s41467-023-38432-6)
Supplement: Supplementary file 3 — Description of Additional Supplementary Informaation [file 41467_2023_38432_MOESM3_ESM.pdf]

Supplementary Data 1: Summary of patient demographics and data available for each sample

Supplementary Data 2: Table of all mutations identified in whole exome sequencing

Supplementary Data 3: List of differentially expressed genes

Supplementary Data 4: Computational drug screen results for MPNST-G1 and MPNST-G2

Supplementary Data 5: Table of all gene fusions identified in tumor cohort
