# Supplementary material for: Multiplatform molecular profiling uncovers two subgroups of malignant peripheral nerve sheath tumors with distinct therapeutic vulnerabilities
Source: Nat Commun. 2023 May 10;14:2696. doi: 10.1038/s41467-023-38432-6 (PMC10172395; doi:10.1038/s41467-023-38432-6)
Supplement: Supplementary file 9 — Supplementary Figure Legends [file 41467_2023_38432_MOESM9_ESM.docx]

**Supplementary Figure 1: Methylation profiling identifies two distinct groups of MPNSTs.** Unsupervised consensus hierarchical clustering of the 20,000 CpGs that show the highest median absolute deviation across the β values in the (a) Toronto cohort (b) TCGA cohort, (c) DKFZ cohort and (d) combined cohort. The change in the cumulative distribution function plot and silhouette analysis plot are shown for each iteration of clustering.

**Supplementary Figure 2: Promoter CpG Island Hypermethylation Phenotype in MPNST-G1. (a)** Number of unique and overlapping probes that are differentially methylated (FDR corrected p-value <0.05 and mean β value difference > 0.1) when comparing subgroups to each other. Boxplots showing the odds ratio and 95% confidence interval for differentially methylated probes (n = 108 samples) based on epigenetic substructures (b) and gene regions (c). Data are presented as mean values +/- SEM. Volcano plots for (d) DKFZ and (e) TCGA cohorts comparing the number of significant methylated probes of CpG islands in the promoter region between MPNST-G1 and MPNST-G2 (FDR corrected p value < 0.05 and mean β value difference > 0.1).

**Supplementary Figure 3 – Validation of PRC2 mutations in MPNST-G1 in TCGA dataset.** Results from whole exome sequencing of (a) neurofibromas (n = 37) in the Toronto cohort with frequency and mutations identified. (b) t-distributed stochastic neighbor embedding (tSNE ) of methylation profiles of all neurofibromas and a previously published cohort of Schwannomas. All neurofibromas with LZTR1 or NF2 mutations are marked as black dots. (c) Results from whole exome sequencing of MPNSTs (n = 5) in the TCGA dataset with frequency and mutations identified.

**Supplementary Figure 4: Transcriptome characterization validates the robustness of molecular subgroups of MPNSTs.** Unsupervised consensus hierarchical clustering of the 250 genes that show the highest median absolute deviation in expression values (a) Toronto cohort and (b) TCGA cohort. The change in the cumulative distribution function plot and silhouette analysis plot are shown for each iteration of clustering. (c) Overlap analysis between the independent cohort clustering (panel a+b) and methylation-based classification. The degree overlap was analyzed using the adjusted Rand index. (d) Unsupervised clustering of tumors using SHH and WNT pathway related genes.

**Supplementary Figure 5: NF1 pathway is inactivated in MPNST-G1 and MPNST-G2 tumors** (a) Boxplot of the distribution of expression of *NF1* gene in the methylation-based subgroups. Show the median, first and third quartiles (boxes), and the whiskers encompass the 1.5X the interquartile range. One-way ANOVA (p < 0.01). Comparison of pathway activity in MPNST-G1 compared to MPNST-G2 tumors, with respect to (b) RAS pathway downstream of NF1, (c) RAF pathway and (d) MEK pathway. GSEA plots for each pathway shown. (e) Representative IHC for NF1 expression in tumor subtypes. (f) Quantification of NF1 expression (n = 23 samples). Data are presented as mean values +/- SEM.

**Supplementary Figure 6: WNT pathway is overexpressed in MPNST-G2 tumors.** GSEA plots for pathway activity of WNT, β-catenin and Cyclin D1 pathways in (a) MPNST-G1 and (b) MPNST-G2 tumors. (c) Tumors were stained by immunohistochemistry for SMO and Beta-Catenin (n=23).

**Supplementary Figure 7:** **SHH and WNT pathway genes expression are correlated with methylation status.** (a) Correlation of gene expression and methylation of promoter region (using all probes in the promoter) for key SHH and pathway genes. Pearson’s correlation. (b) Venn diagram depicting alterations in key SHH pathway genes for MPNST-G1 and MPNST-G2 tumors.

**Supplementary Figure 8: Gene fusion landscape of the spectrum of peripheral nerve sheath tumors.** CIRCOS plot showing the landscape of gene rearrangements detected in (a) MPNST-G1, (b) MPNST-G2, (c) premalignant neurofibroma-G3, (d) benign neurofibroma-G4 and (e) non-syndromic spinal neurofibroma-G5. (f & g) Genomic view of JARID2-ATP5MC2 fusions seen in two MPNST-G1 samples. From the top showing the gene loci within the chromosomes, the number of discordant (split and spanning) reads supporting breakpoint (curved red line), annotated exons of fusion partner genes, and plots of the RNA read counts along the genomic coordinates for the fusion partners in mega basepairs from p-telomere of chromosome 12.

**Supplementary Figure 9: MPNSTs show low within patient variation of copy number profile.** (a) t-SNE representation of the snRNA-seq dataset. (b) Inferred genome-wide CNVs of single nuclei of immune cells (top panel), and neoplastic cells (bottom panel). Sample and cluster annotations are shown on the left.

**Supplementary Figure 10: Characterization of non-tumoral cells within MPNSTs.** (a-d) Uniform manifold approximation and projection (UMAP) of the single cell dataset with tumor samples (b), MPNST subgroup (c) and cell type (d) labelled. (e-h) Heatmap of markers for macrophages (e), endothelial cells (f), T-cells (g) and B-cells (h).

**Supplementary Figure 11: Harmony Batch Correction.** tSNE and UMAP representation of single cell data after HARMONY batch correction

**Supplementary Figure 12: MPNST-G2 tumors demonstrate higher immune cell infiltration** (a) tSNE plot of the snRNA-seq dataset, with clusters annotated by cell type. (b) Bar plot depicting the cell composition of each tumor. (c) Boxplot showing the distribution of LUMP scores for each subgroup in Toronto cohort (n = 108 samples). One-way ANOVA (p < 0.0001). (d) Boxplot showing the distribution of LUMP scores for each subgroup in DKFZ cohort (n = 33 samples). Student T-test, two-sided (p < 0.0001) (e) Boxplot showing the immune score distribution for each subgroup in Toronto cohort (n = 108 samples). One-way ANOVA (p = 0.02) All boxplots show the median, first and third quartiles (boxes), and the whiskers encompass the 1.5X the interquartile range. (f) Tumors were stained by immunohistochemistry for macrophages (CD68).

**Supplementary Figure 13: snRNAseq demonstrates MPNST-G1 and MPNST-G2 tumoral cells are distinct from each other** (a) Boxplot depicting the distribution of correlation scores for tumoral cells in each tumor with bulk transcriptional signatures for each subgroups (n = 30,518 nuclei). (b-d) Boxplot showing the expression of *SMO*, *PTCH1* and *WNT11* genes in tumoral cells within each subgroup (n = 30,518 nuclei). (e) Boxplot showing the predicted neurofibroma cell composition in each subgroup. All boxplots show the median, first and third quartiles (boxes), and the whiskers encompass the 1.5X the interquartile range. A neurofibroma cell signature was generated from the single cell data, and CIBERSORT was used to predict neurofibroma cell composition from the bulk RNA seq data (n= 49 samples). (f) Pairwise correlations between the expression profiles of single tumoral cells from all 6 tumors.

**Supplementary Figure 14: MPNST-G2 resemble Schwann-cell precursor cells.** (a) Boxplots showing the expression of key neural-crest/Schwann cell lineage markers for tumoral cells in each subgroup (n = 30,518 nuclei). Show the median, first and third quartiles (boxes), and the whiskers encompass the 1.5X the interquartile range. (b) tSNE representation of the single cell dataset. Tumoral cells are overlaid with correlation scores with the Schwannoma signature. (c) Boxplot showing the distribution of correlation scores for tumoral cells in each subgroup (n = 30,518 nuclei). Show the median, first and third quartiles (boxes), and the whiskers encompass the 1.5X the interquartile range. (d) Tumors were stained by immunohistochemistry for Schwann cell (S100B) and Schwann cell precursor (GAP43) markers. (e) Trajectory analysis with pseudotemporal continuum plotted. Panel on left annotates the sample of origin and panel on right annotates the tumor subtype on each cell along the pseudotemporal continuum. (f) Heatmap of neural crest cell, Schwann cell precursor cell and Schwann cell markers.

**Supplementary Figure 15: Methylation profiling of MPNST cell lines confirm 2 subgroups (**a) 4 MPNST cells lines were screened for WNT/CCND1 pathway activation. Error bars, s.e.m; n=3 biologically independent experiments. (b) tSNE plot depicting the methylation profile of MPNST cell lines and MPNST human tumor samples.

**Supplementary Figure 16: PTCH1 knockdown leads to increased tumor formation.** (a) Table of tumor formation in xenografts injected with HSC1 cells with *NF1* and/or *PTCH1* knockout. (b) Expression of *GLI1*, *GLI2* and *GLI3* in S462TY cell lines treated with sonidegib. Error bars, s.e.m; n=3 biologically independent experiments. (c) Expression of *GLI1*, *GLI2*, and *GLI3* in STS-26T cell lines treated with sonidegib. Error bars, s.e.m; n=3 biologically independent experiments.
